# Harnessing Phase Separation for the Development of High‐Performance Hydrogels

**DOI:** 10.1002/advs.202600032

**Published:** 2026-03-02

**Authors:** Yue Shao, Yiming Ma, Baihao Shao, Molly M. Stevens

**Affiliations:** ^1^ Department of Physiology, Anatomy and Genetics Department of Engineering Science and Kavli Institute for Nanoscience Discovery University of Oxford Oxford UK; ^2^ Department of Materials Department of Bioengineering and Institute of Biomedical Engineering Imperial College London London UK; ^3^ Department of Biomedical Engineering The Chinese University of Hong Kong Hong Kong China

**Keywords:** hydrogel, phase separation, polymer, soft robotics, wearable sensors

## Abstract

Hydrogels are indispensable for the development of next‐generation bioelectronics, soft robotics, and biomedical devices, where their mechanical properties determine performance and reliability. Among strategies to enhance hydrogel mechanics, phase separation enables controlled heterogeneity resulting in gel networks that are reinforced by more than just covalent bonds and polymer entanglements. By regulating the demixing of polymer‐rich and solvent‐rich domains, phase separation leads to architectures that couple strength, elasticity, and dynamic responsiveness. This article reviews the recent advances in designing high‐performance phase‐separated hydrogels by linking phase separation behavior within polymer networks to emergent properties such as toughness, fatigue resistance, adhesion, and stimuli‐responsiveness. We highlight how mesoscale organization governs multifunctional performance and demonstrate how these principles help resolve the key trade‐offs in critical applications, such as high‐pressure hemostatic sealants, low‐impedance bioelectronics, perfusable tissue engineering scaffolds, and adaptive soft robotics. Finally, we discuss critical challenges, including in situ characterization and scalability, and future opportunities like machine‐learning‐guided design, which are essential to translate phase separation from a materials heuristic into design rules for reliable, high‐performance hydrogel materials.

## Graphical abstract

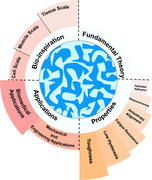

## 1 Introduction

Hydrogels are a unique class of soft materials composed of hydrophilic polymer networks that are swollen with large amounts of water [1]. Their high water content, tuneable elasticity, and excellent biocompatibility make them indispensable in a wide range of applications, including tissue engineering [2, 3, 4], soft robotics [5, 6, 7], bioelectronics [8] and electronics [9, 10], and drug delivery [11]. The ability of hydrogels to combine solid‐like mechanical integrity with liquid‐like permeability enables them to serve as artificial analogues of biological tissues [12]. Despite their versatility, conventional homogeneous hydrogels remain mechanically weak, brittle, and functionally limited [13, 14]. Their uniform network structure leads to stress concentration and irreversible fracture under load. Furthermore, conventional hydrogels often lack hierarchical and multiphase structures which are essential for the dynamic and adaptive behavior of most living tissues [15]. Designing hydrogels that are tough, fatigue‐resistant, and adaptive to external environments has therefore become one of the most persistent challenges in materials science.

Natural tissues achieve exceptional mechanical performance through hierarchical and multiphase architectures, in which reversible‐sacrificial bonds, hydrated interfaces, and phase domains cooperatively bear and relax stress. Inspired by these biological strategies, research has sought to construct hydrogels with built‐in microstructural contrast, such as double network systems [16, 17], nanocomposite reinforcements [18] and interpenetrating networks [19]. These advances illustrate that the exceptional mechanical strength in hydrogels arises not from network uniformity but from the cooperative interactions between distinct structural components. Recently, controlled phase separation has emerged as an effective and versatile strategy to engineer such cooperative heterogeneity [20]. Phase separation is a spontaneous demixing process in which an initially uniform polymer solution or reactive system separates into polymer‐rich and solvent‐rich regions due to thermodynamic instability [21]. This instability results from a balance between enthalpic interactions (e.g., polymer–solvent incompatibility) and the entropy of mixing. When this process occurs concurrently with network formation and the demixing is arrested by crosslinking or crystallization, a stable heterogeneous structure can be obtained [22]. The resulting hydrogel contains distinct yet interconnected domains with different compositions, moduli, and water contents, all of which remain mechanically interconnected [23, 24]. Such internal heterogeneity is the key to bridging molecular design and macroscopic performance. The polymer‐rich domains provide load‐bearing strength and toughness, while the solvent‐rich domains contribute flexibility and reversible energy dissipation [23]. The cooperative deformation between these phases redistributes stress, delays crack propagation and enhances toughness. By controlling the thermodynamics and kinetics of demixing, phase separation can be rationally engineered to deliver specific functionalities.

**FIGURE 1:**
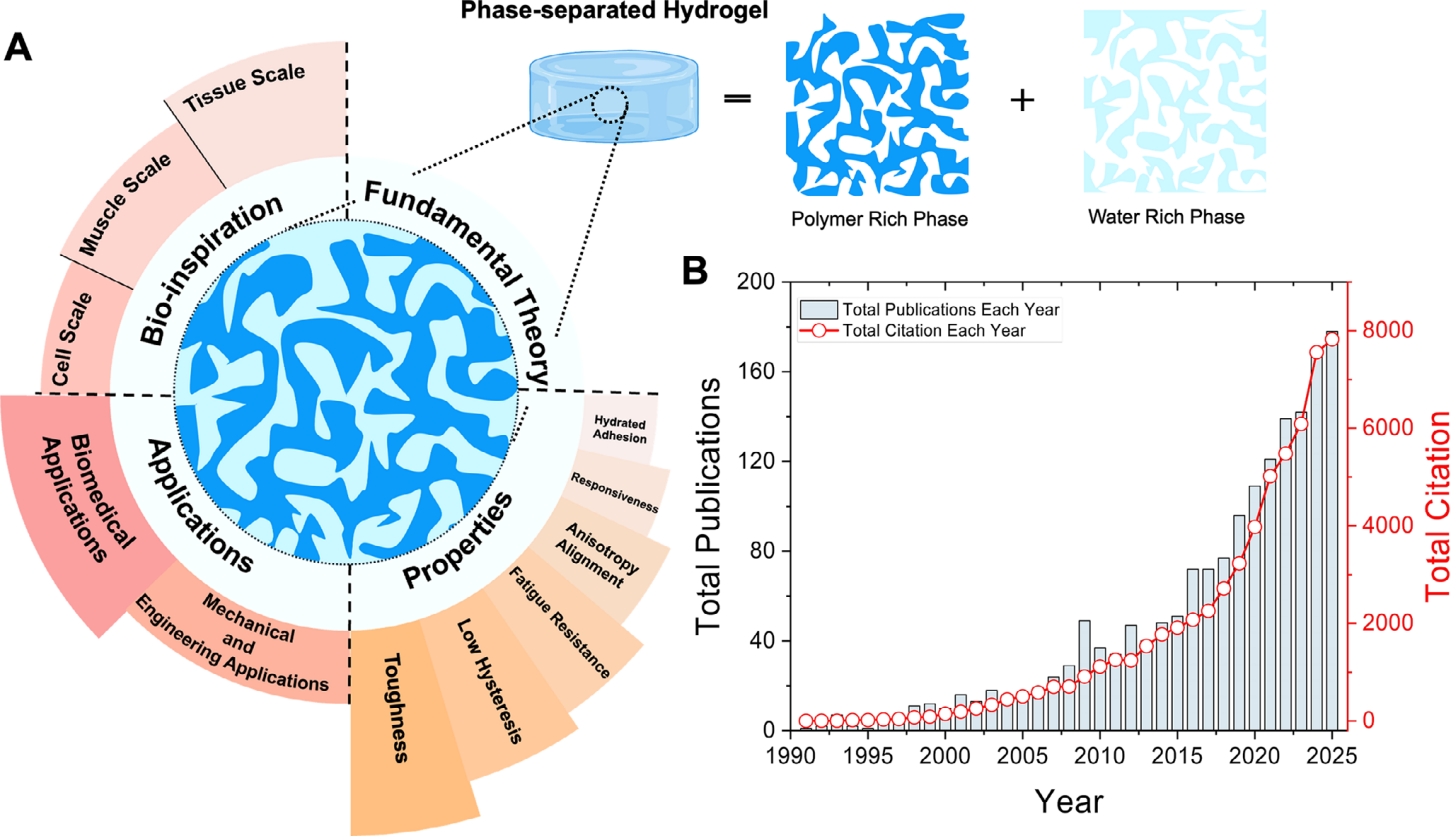
Conceptual framework and research trajectory of phase‐separated hydrogel. (A) Schematic illustration shows the bio‐inspired design, fundamental theory, key properties, and applications of phase‐separated hydrogels. (B) Bibliometric trends demonstrating the field's rapid growth and increasing impact, based on publication and citation data retrieved from the Web of Science database (accessed on January 16, 2026).

While several previous reviews have summarized specific aspects of phase separation in soft materials, they have typically focused either on the thermodynamic fundamentals [25, 26, 27, 28, 29] or on specific applications [30, 31, 32, 33, 34, 35, 36, 37]. A recent review has emphasized preparation strategies and functions of phase separation in polymer gels [20], while another contribution has focused on specific interactions to generate phase‐separated gels [15]. In this review, we aim to provide a rational guideline for the design of high‐performance hydrogels through controlled phase separation. We begin with an overview of the fundamental thermodynamic mechanisms that govern phase separation in polymer‐solvent and polymer‐polymer systems, highlighting the interplay between mixing enthalpy, entropy, and network elasticity as described by the Flory‐Huggins and Flory‐Rehner theories [38, 39]. We then discuss how different phase‐separation pathways, including polymerization‐induced, solvent‐induced, thermally induced, and liquid–liquid demixing, give rise to characteristic morphologies ranging from droplet‐matrix to bicontinuous structures. Particular emphasis is placed on how these mesoscale organizations dictate key mechanical properties (e.g., toughness, low hysteresis, fatigue resistance, and anisotropy) and how they impart responsive behaviors such as thermal switching and underwater adhesiveness for applications in biomedical robotics. Furthermore, we highlight how phase separation is harnessed to create porous, extracellular matrix (ECM)‐mimicking architectures that enhance cellular infiltration in tissue engineering and how it can be leveraged to generate continuous conductive networks that improve electrical signal transduction in bioelectronic devices. Finally, we summarize recent advances in key applications of phase‐separated hydrogels, with special attention to their biomedical and engineering relevance (Figure 1A). This expanding utility has catalyzed a surge in research activity, evidenced by the rapid accumulation of literature (Figure 1B), which underscores the need for a systematic consolidation of the field. Through this review, we seek to connect fundamental understanding with recent experimental progress and establish general design principles for high performance phase‐separated hydrogels.

## 2 General Concept of Phase Separation and its Relevance to Hydrogels

Phase separation is a ubiquitous phenomenon in soft matter, where a uniform mixture spontaneously demixes into coexisting domains due to unfavorable enthalpic interactions and entropic effects. These processes result in characteristic morphologies ranging from droplet‐matrix structures to bicontinuous interpenetrating networks. In hydrogel synthesis, phase separation can be triggered during the polymerization of one component. As predicted by the Flory‐Huggins theory, the miscibility in dilute and semi‐dilute macromolecular solutions varies with chain length as the critical interaction parameter for demixing (χ) depends inversely on the degree of polymerization (N). By tuning χ and N, phase separation can proceed along two distinct pathways, i.e., binodal demixing, which results in droplet‐matrix morphologies, and spinodal decomposition that leads to bicontinuous structures. In hydrogels, the kinetic arrest of these processes by crosslinking locks in heterogeneous architectures that strongly influence the material's mechanical and functional properties. In this section, we first highlight biological inspirations where nature leverages phase separation to achieve specific functions. We then discuss phase separation in soft‐matter physics with an emphasis on thermodynamic driving forces, morphologies, and the distinction between dynamic and arrested states. Finally, we outline the crucial role of phase separation in hydrogels, emphasizing how it provides internal structural control distinct from homogeneous gels or filler‐based composites.

### 2.1 Biological Inspirations

**FIGURE 2:**
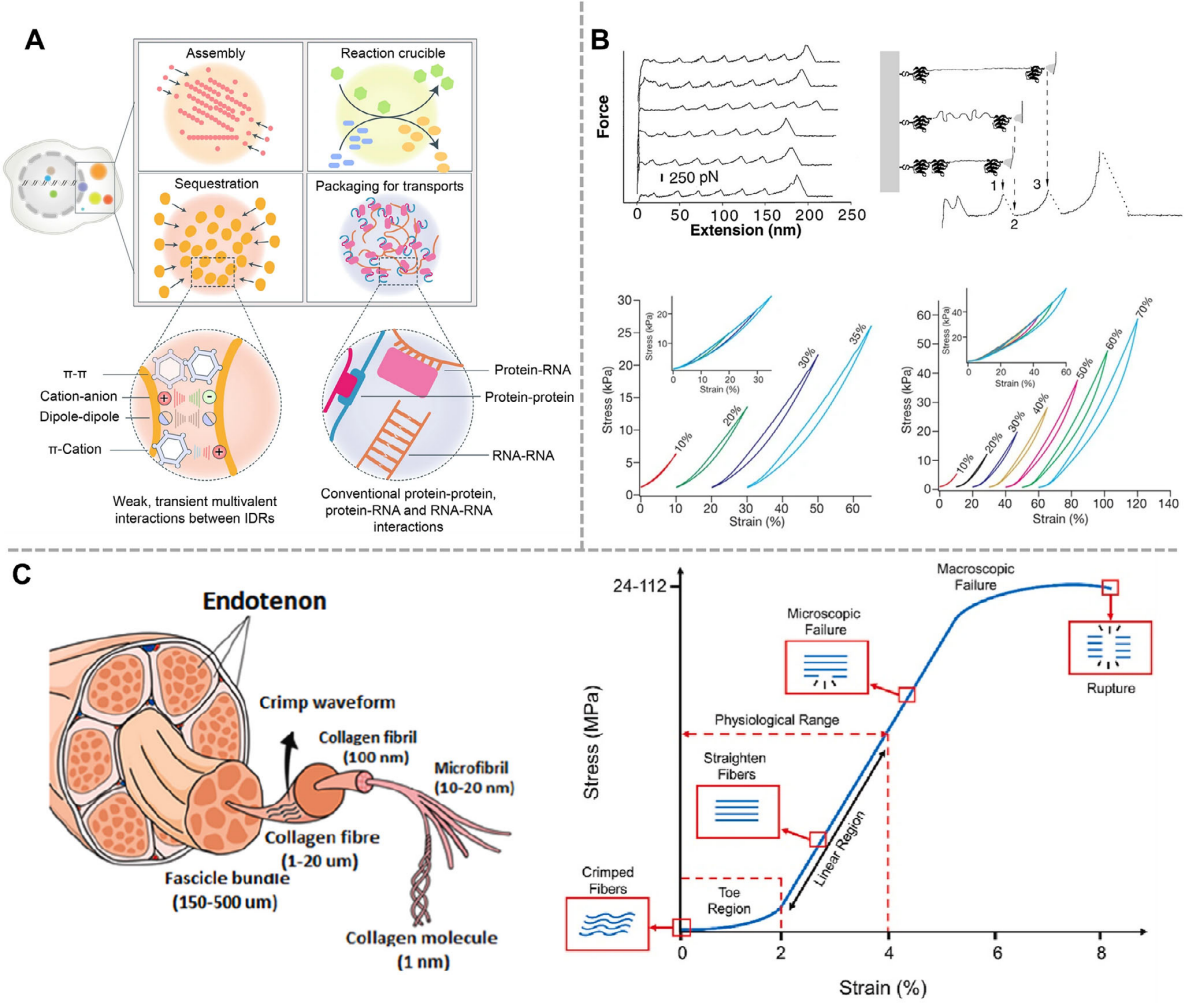
Biological inspiration and hierarchical phase separation in nature. (A) Biomolecular condensates function in complex assembly, reaction facilitation, sequestration, and transport. LLPS arises from multivalent interactions, including conventional protein‐protein/RNA interactions and weak transient interactions among intrinsically disordered regions (IDRs) (π–π, cation–anion, dipole–dipole, π–cation). Reproduced under terms of the CC‐BY license [59]. Copyright 2021, The authors, published by Springer Nature. (B) (Top) Single‐molecule force‐extension curves of recombinant titin segments (Ig8). The characteristic “sawtooth” peaks correspond to the sequential unfolding of individual Ig domains under tension, a mechanism that dissipates energy while allowing significant extension [53]. Reproduced with permission. Copyright 1997, American Association for the Advancement of Science. (Bottom) Macroscopic stress‐strain curves of biomimetic protein hydrogels engineered from repeating polypeptide sequences (G‐R)4 (left) and GRG5RG4R (right). These materials replicate the molecular unfolding mechanism on a macro‐scale. Insets: Cyclic loading curves showing superposition at various strain levels, demonstrating the material's ability to reversibly recover its structure after deformation. Reproduced with permission [54]. Copyright 2010, Springer Nature. (C) The hierarchical structure of tendon and ligament and the corresponding stress‐strain curve illustrating collagen fibre behavior. Reproduced under terms of the CC‐BY license [60]. Copyright 2023, The authors, published by Elsevier.

One classic example of phase separation in biological systems is liquid–liquid phase separation (LLPS). This phenomenon is governed by two distinct thermodynamic formation modes: segregative and associative [40]. Segregative LLPS arises from mutual repulsion between different macromolecular components. A common example is the polyethylene glycol (PEG)/dextran (DEX) system, where mutual incompatibility propels their segregation into an aqueous two phase system (ATPS), consisting of one phase enriched with PEG and the other with dextran [41]. Similar ATPS can also be formed by polymer‐salt repulsions [42]. In contrast, associative LLPS, more commonly termed coacervation, is driven by attractive non‐covalent interactions. These forces compel different (macro)molecules to complex and condense, resulting in a solute‐rich coacervate phase that coexists with a solute‐depleted supernatant [43]. The molecular attractions are driven by a series of non‐specific interactions such as electrostatic forces, hydrogen bonding, and hydrophobic or π‐π stacking, as well as highly specific recognition events like nucleic acid base pairing and protein recognition [44, 45, 46, 47]. As a result, the stability of both ATPS and coacervate systems is highly sensitive to environmental factors like pH, ionic strength, and temperature, which disrupt these associations [48, 49, 50]. At the cellular scale, this associative mechanism is fundamental. LLPS underlies the formation of membraneless organelles by organizing proteins and RNAs into dynamic condensates without enclosing membranes [43, 46]. This process is driven by weak, multivariable interactions, often mediated by intrinsically disordered regions and modular sticker and spacer motifs (Figure 2A). These interactions cause the demixing of a biomolecule‐rich condensed phase from a surrounding dilute phase. The material state and internal dynamics of these condensates span a continuum from liquid‐like to solid‐like states, encompassing various gel‐like and glassy states, depending on complex factors like composition, valency, macromolecular crowding, ionic strength, pH, temperature, and time‐dependent maturation [47]. Condensates display finite interfacial tension, coalescence, and continuous exchange of components with the milieu, which together enable selective partitioning and regulation of reaction rates in highly hydrated environments. By concentrating and segregating specific factors, phase‐separated droplets function as dynamic microreactors that support processes such as signal transduction, the assembly and selective transport associated with the nuclear pore permeability barrier, and transcriptional and post transcriptional control including mRNA processing and localization [51, 52].

In addition to intracellular phase separation, muscle provides a salient paradigm for how internal heterogeneity governs mechanical function at the molecular scale. Its passive response is compliant and resilient at small strains yet progressively dissipative at larger strains, largely due to the I band of titin, where folded immunoglobulin (Ig)‐like domains are interspersed with intrinsically disordered segments (Figure 2B) [53, 54]. Folding of an Ig domain is driven primarily by the hydrophobic effect, in which burial of nonpolar side chains stabilizes a compact, ordered core relative to the solvated, disordered state [55]. Applied force changes this free energy landscape, which lowers the barrier to unfolding, increases contour length, and dissipates mechanical work. Upon unloading, domains often refold on short timescales, restoring elasticity. Although this is not phase separation in the classical multichain sense, the folded versus disordered partitioning is a molecular scale analogue of phase separation in which two mechanically distinct states coexist and interconvert under load, leading to reversible transitions between a condensed, stiff state and a compliant, solvent‐rich state. The alignment of sarcomeric assemblies also imparts anisotropy to muscle, with greater stiffness along the fiber axis.

At the tissue scale, tendon exemplifies a hierarchical multiphase composite in which stiff type I collagen fibrils bear load (Figure 2C). These fibrils are embedded within a hydrated, proteoglycan‐rich matrix that provides compliance, with graded mineralization at the enthesis [56, 57]. The mechanical response follows a characteristic stress‐strain curve governed by this architecture: initially, the “toe region” arises from the macroscopic straightening of crimped fibrils within the proteoglycan matrix, allowing for low‐stiffness extension. As strain increases, the fibrils align coherently along the loading axis to enter a linear, high‐stiffness region where the collagen network bears the primary tensile load. At the nanoscale, collagen triple helices and associated peptide motifs can undergo unfolding or slippage of intermolecular bonds assisted by limited force, providing sacrificial dissipation [58]. At the microscale, crimp straightening and fibril‐to‐fibril sliding within the proteoglycan matrix redistribute stress and blunt cracks. The coherent alignment of collagen along the loading axis produces pronounced anisotropy, while the bicontinuous arrangement of stiff and soft phases promotes efficient load transfer with modest hysteresis when interfacial adhesion is high. This multi‐scale and multiphase organization is functionally equivalent to a phase‐separated architecture in which a continuous stiff network interpenetrates a hydrated soft domain. This structure accounts for tendon's combination of tensile strength, resilience, and exceptional fatigue resistance.

### 2.2 Phase Separation in Soft Matter

Phase separation in soft matter is the transformation of a single homogeneous phase into two or more coexisting phases that differ in composition and structure. In polymeric systems the outcome is governed by the competition between mixing entropy and interaction enthalpy, as well as by the kinetics through which a system crosses its phase diagram. The process generates internal length scales from nanometers to micrometers and gives rise to distinct morphologies such as droplet‐matrix dispersions, bicontinuous networks, and hierarchical architectures with multiscale characteristics. In materials, the developing morphology can be latter arrested and preserved within a stable network by gelation, which is central to harnessing phase separation to engineer improved hydrogels.

#### 2.2.1 Thermodynamic Driving Force

The classical description of polymer mixing is provided by the Flory‐Huggins free energy of mixing, which quantifies the Gibbs free energy of mixing (Δ*G_mix_*) for polymers and solvents confined in a lattice model (Equation 1),

(1) $$\;\frac{\Delta G_{mix}}{RT}=\frac{\phi_{s}}{N_{p}}\ln\phi_{p}+\phi_{s}\ln\phi_{s}+\chi \phi_{p}\phi_{s}$$

where *R* is the absolute gas constant, *T* is temperature, ϕ_*p*_ and ϕ_*s*_ are the polymer and solvent volume fractions, *N_p_* is the degree of polymerization, and χ is the Flory‐Huggins interaction parameter, which quantifies the enthalpy (energy) of mixing between a polymer and a solvent (or between two different polymers). For polymers, the entropy of mixing ($\frac{\phi_{s}}{N_{p}}\ln\phi_{p}$) is negligible because of the large *N_p_*, meaning the enthalpic term χϕ_*p*_ϕ_*s*_ dominates phase behavior. As a result, even small unfavorable changes in interaction enthalpy are sufficient to overcome the modest entropy gain, driving demixing and the emergence of compositionally distinct domains. Molecular interactions such as hydrogen bonding, hydrophobic interactions, ionic interactions, dipole‐dipole forces, or donor‐acceptor interactions therefore determine whether phase separation occurs and the strength of interactions between polymer chains in turn affects the enthalpic term. Additionally, the presence of solvent can induce the formation of solvation shells around the polymer chains, leading to further changes in the enthalpic term. A negative χ (typically within ‐0.1 to 0) represents a strong solvation shell, indicating favorable mixing through strong attractive interactions [61]. However, when χ becomes positive, it reflects weakened interactions which can cause phase separation. For polymer‐solvent systems, a Flory‐Huggins interaction parameter χ < 0.5 typically corresponds to good solvent conditions, under which favorable polymer‐solvent interactions promote homogeneous mixing. In contrast, χ > 0.5 indicates increasingly unfavorable polymer–solvent interactions that thermodynamically promote demixing and phase separation. Phase separation occurs when the enthalpic term becomes less favorable as the interactions between the polymer and solvent weaken. As an example, long alkyl chains (C18) were deliberately incorporated into hydrogels to promote hydrophobic associations [62]. The introduction of these hydrophobic moieties increased the effective χ parameter with water, which drove microphase separation into hydrophobic domains.

It should be noted that, in real hydrogels, polymer chains are covalently or physically crosslinked, introducing an elastic free‐energy penalty when the network deforms during swelling or demixing. The Flory‐Rehner theory extends the Flory‐Huggins model by adding an elastic term (Δ*G_elastic_*) representing the restoring force of the network (Equation 2) [63]. The total free energy per mole of lattice sites (Δ*G_total_*) is described as:

(2) $$\Delta G_{total}=\Delta G_{mix}+\Delta G_{elastic}$$

**FIGURE 3:**
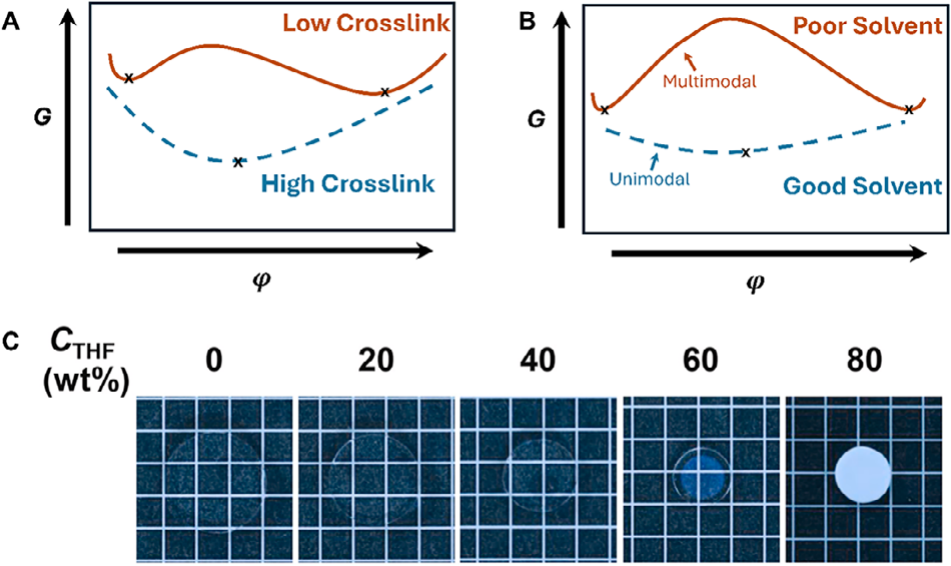
The impacts of crosslink density and solvent on phase separation in polymer networks. Free energy landscapes of polymer gels depending on the crosslinking nature (A) and solvent type (B). The x‐axis represents the polymer volume fraction and y‐axis represents the Gibbs free energy. (C) Increasing the ratio of non‐solvent induces dramatic changes in hydrogel volume, optical properties, and polymer volume fraction. Reproduced with permission [64]. Copyright 2020, Elsevier.

The elastic free energy for an isotropically swollen network is expressed as (Equation 3):

(3) $$\frac{\Delta G_{elastic}}{RT}=\frac{1}{2}\;v\;\left[\;{\left(\frac{\phi_{p}}{\phi_{p0}}\right)}^{\frac{1}{3}}-\;\frac{1}{2}\;\left(\frac{\phi_{p}}{\phi_{p0}}\right)\right]$$

where *v* is the number of network chains per unit volume (i.e., crosslink density), ϕ_*p*_ and ϕ_*p*0_ are polymer volume fractions in the swollen and reference (dry) states, respectively. At swelling equilibrium, the osmotic pressure arising from mixing equals the elastic restoring pressure, leading to the final Flory‐Rehner equation (Equation 4):

(4) $$\ln\left(1-\phi_{p}\right)+\phi_{p}+\chi {\phi_{p}}^{2}+\frac{vV_{s}}{N_{A}}\;\left[\;{\left(\frac{\phi_{p}}{\phi_{p0}}\right)}^{\frac{1}{3}}-\;\frac{1}{2}\;\left(\frac{\phi_{p}}{\phi_{p0}}\right)\right]=0\;$$

where *V_s_* is the solvent molar volume and *N_A_* is Avogadro's number. In this regard, the degree of crosslinking in gels can have an impact on the density *v*. Increasing *v* steepens the elastic free‐energy curve and suppresses large‐amplitude composition fluctuations, stabilizing a more homogeneous phase (Figure 3A). Conversely, networks with lower crosslink densities permit greater chain mobility and thus facilitate phase separation. High crosslink density can therefore hinder demixing by constraining the configurational freedom of polymer chains, while loosely crosslinked or physically associated systems (e.g., hydrophobic, ionic, and H‐bonded gels) more readily form microphase‐separated structures.

Alternatively, χ also changes sharply in response to a non‐solvent penetrating the gel, triggering phase separation (Figure 3B). The introduction of a non‐solvent reduces polymer‐solvent affinity while promoting interchain associations, shifting the thermodynamic balance toward demixing. For instance, immersing hydrophilic copolymer networks in miscible non‐solvents such as acetone and ethanol led to rapid desolvation and thus microphase formation (Figure 3C). The local increase in χ drove polymer aggregation and arrested coarsening through physical crosslinking, resulting in tunable porosity and mechanical reinforcement [64].

#### 2.2.2 Copolymerization‐Induced Phase Separation

Copolymers consist of two or more chemically distinct monomer units covalently joined along the same backbone. The combination of segments with differing affinities toward one another or toward the solvent provides a natural thermodynamic pathway for phase separation. Unlike polymer‐solvent or polymer‐polymer blends, where incompatibility manifests between independent chains, copolymerization embeds this incompatibility intramolecularly. As a result, copolymer‐based phase separation occurs on nanometer to micrometer scales, giving rise to ordered or disordered morphologies that can be precisely controlled through chemical composition, sequence length, and architecture.

**FIGURE 4:**
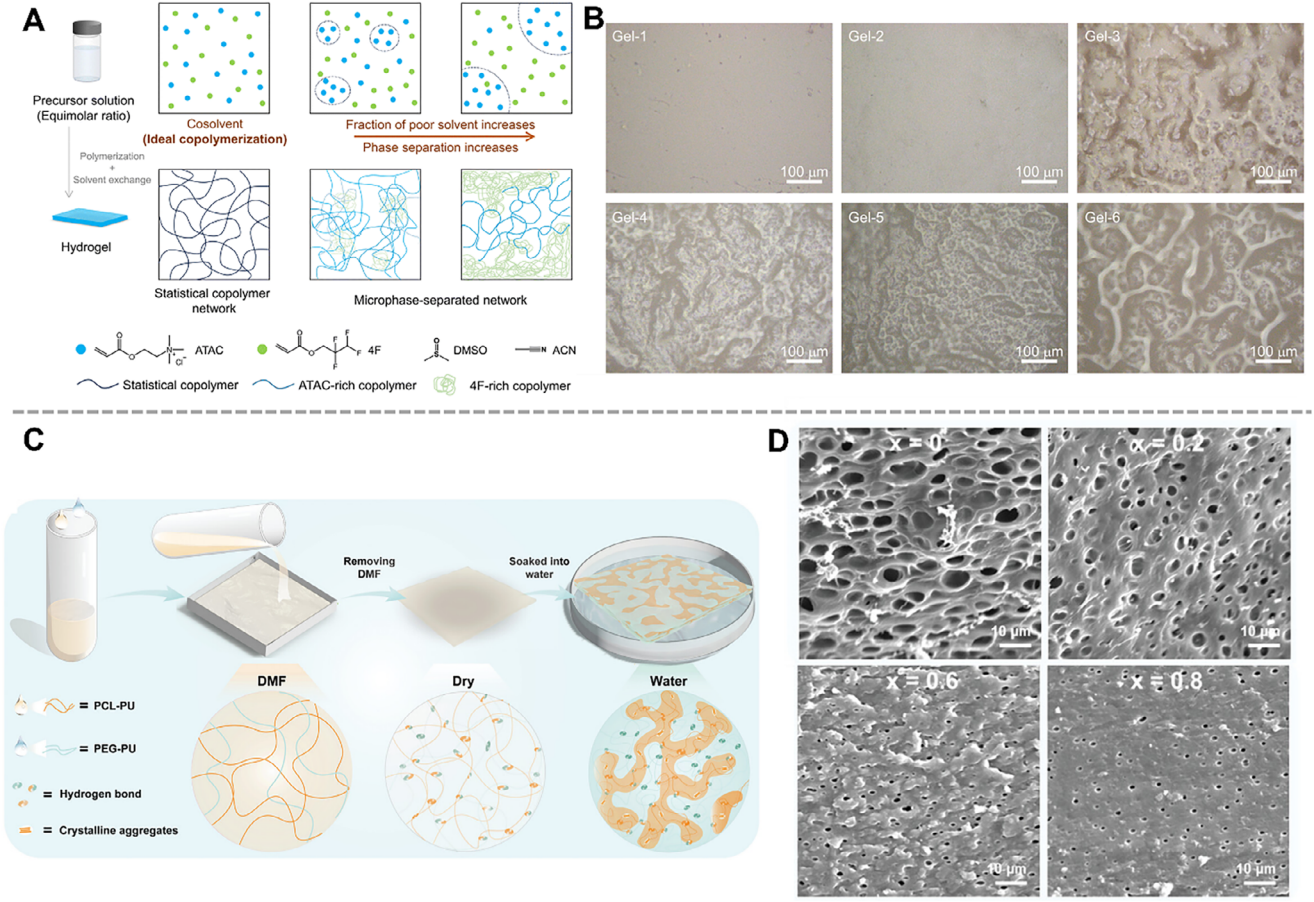
Copolymer and blend‐induced phase separation morphologies. (A) Design strategy for synthesizing hydrophobic copolymer hydrogels with varied monomer distributions and network structures by tuning solvent conditions during free‐radical copolymerization and solvent exchange. (B) Laser microscopy images of hydrogel cross‐sections equilibrated in 0.7 M NaCl. Reproduced under terms of the CC‐BY license [67]. Copyright 2024, The authors, published by the Royal Society of Chemistry. (C) Fabrication of PEG‐PU/PCL‐PU hydrogels featuring a hydrogen‐bonded bicontinuous phase structure. (D) SEM images of PEG‐PU/PCL‐PUx hydrogels. Reproduced with permission [69]. Copyright 2024, John Wiley and Sons.

The thermodynamics of copolymer systems are governed by the same Flory‐Huggins framework described in Section 2.2.1, with the addition of an inter‐segment interaction parameter χ_*AB*_ that characterizes the enthalpic incompatibility between two chemically distinct blocks, A and B. For a binary copolymer melt, the free energy of mixing can be described as (Equation 5):

(5) $$\;\frac{\Delta G_{mix}}{RT}=\frac{\phi_{A}}{N_{A}}\ln\phi_{A}+\frac{\phi_{B}}{N_{B}}\ln\phi_{B}+\chi_{AB}\phi_{A}\phi_{B}\;$$

where ϕ_*A*_ and ϕ_*B*_are the volume fractions of the A and B segments, *N_A_* and *N_B_* are their respective degrees of polymerization, and χ_*AB*_ is the segment‐segment interaction parameter to quantify the incompatibility between blocks A and B. Depending on the volume ratio ϕ_*A*_/ϕ_*B*_, the system can self‐organize into different nano/microstructures such as lamellae, cylinders, spheres, or gyroids. In hydrogels, the same principle applies under swollen conditions, where one or both blocks interact with the solvent. The thermodynamic balance among χ_*AB*_, χ_*PS*_ (polymer solvent interactions) and the network elasticity determines whether domains remain microscopic or grow to sub‐micron scales. For example, histidine methacrylamide and methacrylic acid were copolymerized to form a network stabilized by both hydrogen bonding and hydrophobic interactions [65]. During polymerization, the imidazole groups of histidine methacrylamide form hydrogen bonds with carboxyl groups of methacrylic acid, while their hydrophobic backbones aggregate. As a result, the effective χ_*AB*_ of the histidyl copolymer increased during polymerization, which triggered microphase separation of the hydrophobic domains within a hydrophilic matrix. In another example, hydrogen‐bonding hydrophilic (acrylic acid) and more hydrophobic “core‐forming” monomers (diacetone acrylamide) underwent dispersion copolymerization. As conversion increases, the particles jam and interconnect into a percolated network [66]. By varying the total monomer concentration (solid content) and the hydrophobic‐monomer fraction, the phase separation tendency can be rationally manipulated. For instance, decreasing the hydrophobic monomer resulted in a smooth microstructure and attenuated phase separation. In a recent example, Gong and colleagues demonstrated how solvent manipulation led to distinct hydrogel networks from a hydrophobic copolymer (Figure 4A) [67]. In their study, equimolar cationic and fluorous monomers, 2‐(acryloyloxy)ethyl trimethylammonium chloride and 2,2,3,3‐tetrafluoropropyl acrylate, were copolymerized via free‐radical polymerization under varying solvent mixtures of dimethyl sulfoxide (DMSO) and acetonitrile (ACN). The different solubilities of the two monomers in the mixed solvents dictated their local distribution and consequently the effective inter‐segment interaction parameter χ_*AB*_. Under DMSO‐rich conditions, both monomers were completely soluble and copolymerized in a nearly ideal statistical manner, resulting in transparent networks with homogeneous composition. As the proportion of ACN increased, the solubility of the cationic monomer decreased, inducing local concentration fluctuations and phase separation during polymerization. This heterogeneity increased the enthalpic penalty of mixing and raised χ_*AB*_, leading to the formation of discrete hydrophobic domains within a continuous hydrophilic matrix, i.e., heterogeneity. (Figure 4B). The resulting hydrogels exhibited reduced swelling and enhanced stiffness as the microphase‐separated regions acted as reinforcing domains that resisted osmotic expansion.

#### 2.2.3 Polymer‐Blend‐Induced Phase Separation

Polymer‐blend‐induced phase separation refers to demixing in mixtures of two chemically independent polymers (A/B), typically prepared in a common solvent and subsequently arrested by aggregation (physical) or crosslinking (chemical). This process is driven by the thermodynamic immiscibility of distinct macromolecular species already present in the system. The morphology and stability of these blends depend critically on the balance of enthalpic interactions between polymer components, their molecular weights, and the presence of solvents and crosslinkers. In this case, as polymers possess large *N_p_* values, the entropic contribution is minimal, and the miscibility is predominantly governed by the enthalpic terms. Consequently, even a slightly positive χ_*AB*_ can result in blend immiscibility. Therefore, polymer blends are intrinsically prone to phase separation unless favorable specific interactions (e.g., hydrogen bonding and ionic complexation) offset the enthalpic penalty.

A particularly illuminating example of polymer‐blend‐induced phase separation is based on the amphiphilic entangled network design, where semicrystalline poly(lactic acid) (PLA) chains were physically blended into a hydrophilic PEG diacrylate (PEGDA) network to form an interpenetrating hydrogel [68]. Upon swelling in water, phase separation was thermodynamically driven by the hydrophobic PLA segments, which aggregated into semi‐crystalline microdomains while remaining entangled with the PEGDA matrix. This combination of enthalpic segregation (PLA crystallite formation) and entropic entanglement produced a highly heterogeneous yet mechanically robust structure. Small‐angle X‐ray scattering (SAXS) and scanning electron microscopy (SEM) revealed uniform PLA‐rich domains (∼5 µm) dispersed within the hydrophilic network, confirming the microscale arrest of the phase‐separated morphology. In another example, hydrophilic PEG‐based polyurethane (PEG‐PU) was blended with hydrophobic poly(𝝐‐caprolactone)‐based polyurethane (PCL‐PU) to form a dry film (Figure 4C) [69]. The dry film was then swollen to induce microphase separation, and a hydrogen bonding enhancer imidazolidinyl urea (IU) was incorporated into both PU components, allowing hydrogen bonding across the hydrophilic and hydrophobic phases. These hydrogen bonds act to moderate interfacial energy and suppress gross macroscopic separation, enabling the formation of a bicontinuous microstructure of interlocking PEG‐PU and PCL‐PU domains. Notably, the SEM images show a uniform porous structure with a pore size of approximately 6 µm. When the mass fraction of one component increased from 0.2 to 0.8, the pore size decreased because of the increased crosslinking density of the hydrogel network induced by microphase separation (Figure 4D). This work exemplifies how the interplay between entropic swelling and specific supramolecular interactions (e.g., hydrogen bonds) governs not only phase separation but also the extent and fineness of the resulting domains.

#### 2.2.4 Lower Critical Solution Temperature (LCST) and Upper Critical Solution Temperature (UCST) Transitions

**FIGURE 5:**
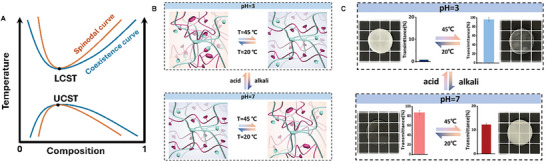
**FIGURE 5** Temperature‐responsive phase separation. (A) Phase diagrams showing LCST and UCST behavior of polymers. The diagram plots Temperature against Polymer Composition. Coexistence Curve (Blue / Binodal): The boundary separating the thermodynamically stable single‐phase solution (mixed) from the two‐phase region. Crossing this line into the gap between the curves enters the metastable region, where phase separation typically proceeds via nucleation and growth (forming droplets). Spinodal Curve (Orange): The boundary of absolute instability. Crossing this line into the unstable region triggers spontaneous, continuous demixing known as spinodal decomposition, which typically results in bicontinuous, interconnected networks. (B) Schematic of pH‐induced reversible temperature response of PACA‐HPC hydrogels exhibiting both LCST and UCST behaviors. (C) Reversible temperature responsiveness of PACA‐HPC hydrogels under acidic and alkali conditions. Reproduced with permission [74]. Copyright 2024, John Wiley and Sons.

In many polymer‐solvent systems, the Flory‐Huggins interaction parameter χ is temperature dependent. Therefore, phase separation can be induced by thermal stimuli via manipulating temperature near the LCST and UCST. These temperature‐triggered transitions can be harnessed in hydrogels to embed thermally controlled (de)mixing behaviors (Figure 5A).

Below the LCST, the polymer is fully miscible with the solvent (single phase). Above the LCST, polymer‐solvent interactions become unfavorable, and the system demixes into polymer‐rich and polymer‐poor phases, which drives phase separation. As temperature increases, the favorable enthalpic contribution is outcompeted by the entropic penalty, making Δ*G_mix_* unfavorable. For example, poly(N‐isopropylacrylamide) (PNIPAM) exhibits an LCST near 32°C in water. As temperature rises, intermolecular hydrogen bonds between water and polymers dissociate, causing the chains to collapse and aggregate. This leads the polymer chains to undergo a coil‐globule transition, dehydration, and aggregation. For instance, a homo‐PNIPAm hydrogel underwent phase separation upon heating above the critical temperature, leading to a more than 10‐time increase in Young's modulus [70]. During phase separation, the PNIPAm chains self‐associate and form a polymer‐rich percolating framework which serves as a load‐bearing phase to increase gel stiffness, extensibility, and toughness by enhancing energy dissipation. In another example, covalently crosslinked PNIPAM/poly(N,N‐dimethylacrylamide) (PDMA) hydrogel networks capable of temperature‐responsive microphase separation under isochoric conditions were developed [71]. In this design, PNIPAM provides the thermoresponsive component with an LCST near 30°C, while PDMA side chains maintain hydration and suppress bulk deswelling, thereby counteracting the macroscopic deswelling effects of phase separation. Small‐angle neutron scattering (SANS) demonstrated the emergence of a distinct correlation peak above 50°C, signifying the formation of PNIPAM‐rich domains with a periodicity of approximately 88 nm and a local polymer fraction of 0.7–0.75. This transition resulted in reversible thermoresponsive behavior, where the shear modulus and fracture energy increased from approximately 1 kPa to 10 kPa and 30 to 1000 J m^−^^2^, respectively, accompanied by low viscoelastic loss and stable cyclic performance at 60°C. Under deformation, PNIPAM‐rich domains oriented along the tensile axis, guiding cracks through tortuous paths and dramatically enhancing energy dissipation.

In contrast to LCST, UCST behavior arises when attractive interchain interactions (i.e., ionic pairing, dipole‐dipole association or hydrogen bonding) are strong at low temperature but are weakened by thermal agitation. In other words, at low temperature, chains prefer to associate (i.e., phase‐separated), but at high temperature, thermal motion overcomes those interactions and yields mixing. Therefore, in the UCST regime, the polymer and solvent are miscible only above a threshold temperature. Cooling below the UCST leads to phase separation (i.e., demixing upon cooling). One representative example of a UCST‐type system is polyzwitterion poly(sulfobetaine methacrylate), where cooling strengthens electrostatic pairing and drives association. In one particular example, zwitterionic sulfobetaine nanocomposite (NC) gels displayed exceptional mechanical toughness and well‐defined UCST‐type thermosensitivity [72]. Thermodynamically, the system derives its responsiveness from the ion‐pairing equilibrium between cationic and anionic moieties on the sulfobetaine unit. At low temperature, these ion pairs aggregated into zwitterionic clusters, promoting polymer‐polymer association and gel opacity. At elevated temperature, the ion pairs dissociated and yielded transparent and swollen hydrogel networks. The UCST could be finely tuned by adjusting the alkyl‐chain length between charged groups (A_3_ ≈ 16°C; A_4_ ≈ 85°C), the comonomer ratio with hydrophilic N,N‐dimethylacrylamide (DMAA), and the clay content. For instance, partial copolymerization with 10 mol % DMAA (A_3_D_10_‐NC_3_) reduced the UCST to ≈ 9°C and increased the tensile strength to ≈ 53 kPa, illustrating how composition controls both thermal response and mechanics. Poly(N‐acryloylglycinamide) is also a well‐established UCST‐type polymer that can be designed into supramolecular hydrogels [73]. The molecular design exploits the dual amide motif of the glycinamide side group, capable of forming bifurcated hydrogen bonds that cooperatively stabilize each other even in aqueous media. The UCST‐type system manifests through reversible association upon cooling and dissociation upon heating. Dynamic mechanical analysis curves show that both the storage and loss moduli decrease markedly as temperature rises from 23°C to 76°C, confirming that the network softens due to the disruption of the hydrogen‐bonded crosslinks. Conversely, the hydrogen bonds reform spontaneously, restoring rigidity and physical crosslink density upon cooling. In another work, a hydrogel with both LCST and UCST behaviors was developed by incorporating poly(acrylic acid‐co‐acrylamide) and hydroxypropyl cellulose (PACA‐HPC) hydrogel (Figure 5B) [74]. Under acidic conditions, protonated carboxyl groups form hydrogen bonds with HPC, yielding UCST behavior, i.e., an opaque gel at low temperature that becomes transparent when heated. Under neutral or basic conditions, these hydrogen bonds dissociate and restore HPC's intrinsic LCST behavior, which is opposite to the UCST one (Figure 5C).

#### 2.2.5 Morphologies of Phase‐Separated Structures

The transition of a homogeneous solution into a multiphase mixture results in distinct microstructural architectures, the specifics of which are governed by the thermodynamic pathways of demixing and the competing kinetics between phase separation and network fixation. These arrested morphologies are the primary origins of the unique properties of phase‐separated hydrogels. The process generates internal length scales ranging from nanometers to micrometers, giving rise to morphologies that can be broadly classified into droplet‐matrix dispersions (binodal decomposition) and bicontinuous networks (spinodal decomposition).

**FIGURE 6:**
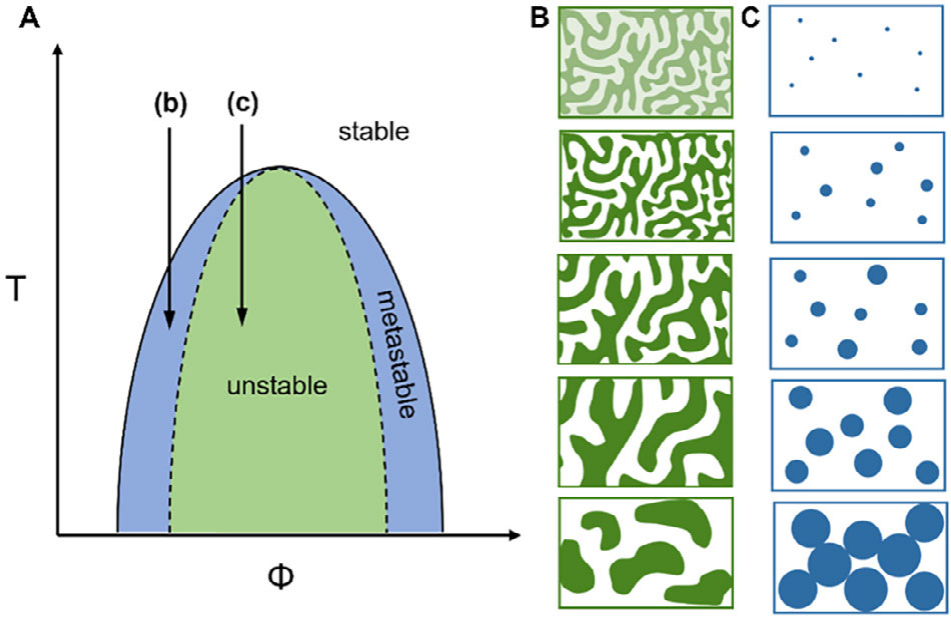
Thermodynamic pathways of demixing. (A) Phase diagram of a binary system showing the binodal (solid) and spinodal (dashed) lines. (B) Schematic of phase separation via spinodal decomposition. (C) Schematic of phase separation via nucleation and growth. Reproduced with permission. [79] Reproduced with permission. Copyright 2023, American Chemical Society.

It is worth noting that the characteristic domain size of phase‐separated structures is quantitatively governed by polymer concentration, solvent quality, and crosslinking density, as well as the relative timescales of demixing and gelation. Decreasing polymer concentration or improving solvent quality reduces solution viscosity and elastic constraints, allowing phase domains to coarsen for longer durations prior to kinetic arrest, thereby yielding larger characteristic length scales. Conversely, higher polymer concentration or poorer solvent quality accelerates demixing while increasing elastic penalties, leading to earlier arrest and smaller domains. Increasing crosslinking density further suppresses coarsening by introducing an elastic restoring force that penalizes composition fluctuations, often confining domain sizes to the nanometer or submicrometer regime [75]. On a phase diagram, the binodal curve (or coexistence curve) defines the temperatures and compositions where phase separation is thermodynamically favorable, separating a single stable phase from a two‐phase region (Figure 6A). The spinodal curve, located inside the binodal curve, marks the limit of local phase stability; within the spinodal, the system is inherently unstable to small composition fluctuations, leading to spinodal decomposition, a continuous and uniform phase separation process (Figure 6B). Between the binodal and spinodal lies a metastable region, where a single phase can exist but requires nucleation to initiate phase separation. In the early stage of spinodal decomposition, domains are initially formed from amplified concentration fluctuations on molecular length scales; they rapidly evolve into an interpenetrating bicontinuous network if the two phases occupy similar volumes. Interfacial tension then drives hydrodynamic coarsening, in which the characteristic domain size increases approximately linearly with time while both phases remain percolated. When the interconnecting ligaments undergo thinning and pinch‐off, the system undergoes the percolation‐to‐cluster transition, after which the domains are physically separated and grow by mass transfer via coalescence or Ostwald ripening. For hydrogels, this coarsening process can be arrested by vitrification, crystallization and gelation. The morphology of the arrested phase domains will significantly influence the mechanical properties of the resulting material. By contrast, binodal decomposition (nucleation‐growth) occurs in the metastable region and proceeds through nucleation and growth, where droplets of the minority phase form and grow within a continuous majority phase (Figure 6C). This results in a classic droplet‐matrix morphology. The final size and distribution of these droplets depend on the competition between the kinetics of phase separation and gelation, the latter of which arrests the coarsening process.

In a representative example, macroporous dextran‐based hydrogels with interconnected pores were prepared by crosslinking methacrylated dextran (DexMA) in the presence of PEG. As DexMA polymerizes, the increasing molecular weight drastically reduces the entropic gain of mixing, rendering the growing chains incompatible with the PEG‐rich phase and driving LLPS. Depending on the kinetic disparity between phase separation and gelation, the system may enter either the binodal (nucleation and growth) or spinodal (continuous demixing) regime of the phase diagram, each producing distinct morphological outcomes [76]. Under a 5 weight percent (wt.%) PEG concentration, the DexMA/PEG/water mixture undergoes spinodal decomposition, leading to an interconnected bicontinuous structure with “interconnected‐beaded” pore walls. This morphology arises because spontaneous composition fluctuations within the spinodal region grow continuously, forming interpenetrating polymer‐rich and solvent‐rich phases before the gel point immobilizes the structure. As a result, the hydrogel exhibits large, interconnected macropores (around 41 µm in diameter) with throats averaging 11 µm. However, if the PEG concentration is too high (> 7 wt.%), the mixture separates rapidly and coarsely, favouring droplet nucleation and coalescence rather than the formation of a continuous spinodal morphology. Using mercury intrusion porosimetry, the authors demonstrated a bimodal pore‐size distribution consisting of macropores with a median diameter of approximately 41 µm interconnected by throats with a median diameter of ∼11 µm, spanning broad ranges of approximately 10–120 µm for pores and 5–20 µm for throats, respectively. In a recent work, the phase separation pathway was shown to be controlled by polymerization kinetics and diffusion rate through viscosity tuning in an aqueous PEG/dextran/hyaluronic acid (HA) system [77]. With high light intensity, the polymerization proceeds rapidly, causing the PEG network to gel before significant coarsening occurs. Under such conditions, the system arrests early in the binodal region, leading to isolated, droplet‐like pores or even nanoporous homogeneous gels rather than a bicontinuous network. In another example, a fully aqueous two‐phase emulsion bioink composed of gelatin methacryloyl (GelMA) and poly(ethylene oxide) (PEO) was developed [78]. When mixed under physiological conditions, GelMA and PEO form a water‐in‐water emulsion where PEO droplets are dispersed in a continuous GelMA‐rich phase. Image‐based analysis of over 100 droplets revealed narrowly distributed pore diameters of 22.7 ± 5.5 µm at a GelMA:PEO volume ratio of 4:1. When the ratio was reduced to 1:1, the diameters increased to 52.7 ± 17.7 µm, reflecting enhanced droplet coalescence prior to photocrosslinking. This demixing proceeds through binodal decomposition with discrete droplets nucleation and growth, followed by GelMA photopolymerization to arrest the phase separation. The dissolution of the free PEO yielded uniform microporous structures.

### 2.3 Why Phase Separation Matters in Hydrogels

Phase separation is a design principle that allows hydrogels to acquire complex, multifunctional architectures from molecularly simple formulations. When a homogeneous polymer solution or prepolymer mixture undergoes demixing during gelation, transient concentration fluctuations become permanently encoded into the solid network as spatially distinct polymer‐rich and solvent‐rich domains. This transformation converts a thermodynamic instability into a functional microstructure [15, 20, 80]. In sharp contrast with homogeneous hydrogels, where stress, ions, and solvents distribute uniformly, the presence of phase‐separated domains introduces heterogeneity in composition, stiffness, and mobility, generating emergent properties that cannot be achieved by uniform crosslinking or by simple filler addition. Arrested phase separation provides a means to “freeze” transient mesoscale order, yielding networks with controlled connectivity and tunable internal length scales that bridge molecular design with macroscopic function [81, 82].

The mechanical performance of hydrogels is one of the clearest demonstrations of how phase separation governs functionality. In bicontinuous morphologies formed through spinodal decomposition, both polymer‐rich and solvent‐rich phases percolate through the material, establishing load‐bearing and dissipative pathways simultaneously [83, 84, 85]. The polymer‐rich domains form a continuous energy‐dissipating skeleton that distributes and bifurcates cracks, while the softer, solvent‐rich regions act as compliant buffers that absorb strain without fracture [86]. In addition, such heterogeneity is often accompanied by remarkable fatigue resistance [87, 88]. Critically, when demixing remains reversible rather than arrested, phase‐separated domains can reversibly form and dissolve under thermal or chemical stimuli, coupling molecular interactions to macroscopic actuation. LCST‐type gels such as PNIPAM leverage temperature‐driven hydrophobic collapse to reversibly modulate modulus, opacity, and solvent uptake [89, 90, 91]. In contrast, UCST‐type zwitterionic systems form and disassemble ionic clusters with temperature, enabling reversible stiffness tuning through the making and breaking of interchain ion pairs [92]. These reversible transitions echo the biological use of LLPS in membraneless organelles, where dynamic partitioning and exchange allow adaptive functions [45, 93]. In hydrogels, the same principle allows the creation of soft matter capable of reversible modulation of mechanical, optical, or transport properties in response to environmental stimuli [46, 94].

Beyond mechanical and responsive behaviors, phase separation critically governs transport, adhesion, and energy dissipation [69, 95, 96]. Bicontinuous morphologies provide percolating solvent‐rich channels that enable rapid ion conduction or molecular diffusion while maintaining mechanical robustness through a polymer‐rich skeleton, which is essential for hydrogels used in bioelectronics, actuators, and tissue scaffolds [77, 97, 98]. Conversely, droplet‐matrix morphologies obtained through binodal decomposition offer localized domains that act as reservoirs or selective barriers, providing tunable permeability and spatial control of chemical reactivity. For instance, in macroporous dextran‐based hydrogels, droplet coalescence followed by gelation yields interconnected pores that facilitate nutrient transport and cell infiltration without sacrificing bulk integrity [77, 84]. Thus, by adjusting the kinetics and extent of demixing, the hydrogel's internal architecture can be rationally tailored to optimize mechanical and transport properties for specific applications. In addition, when phase separation is arrested, the material response is dominated by kinetic processes rather than heat transport. In such systems, shape‐shifting kinetics are dominated by internal chain rearrangement or interfacial reorganization. For example, in shape‐memory hydrogels, heating‐induced phase separation produces a programed, phase‐separated state where polymer chains are packed into condensed polymer domains with limited chain mobility [99]. During natural cooling at ambient temperature, water diffuses into the polymer domains to solvate the polymer chains. Because this redistribution of internal water is a slow, mass transport–limited process, shape recovery is dominated by mass diffusion rather than by heat transport. By altering the degree of phase separation during programming, the recovery onset can be delayed, enabling a naturally triggered shape memory polymer with a tunable recovery onset. Similarly, in thermally induced phase separation ionogels, a metastable polymer‐dense phase is generated during phase separation [100]. After removing the external stimulus, the evolution of the bulk condensed structure and the adsorption of surface droplets proceed with a kinetic difference. The recovery of friction state transition from slippery to sticky is delayed due to the hysteresis between stiffness change and surface transition. By vitrifying the polymer‐dense phase, the metastable intermediate state can be locked, resulting in friction memory that persists after withdrawal of external stimuli. In summary, phase separation provides a powerful framework for translating molecular interactions into macroscopic functionalities in soft materials.

## 3 Key Properties of Phase‐Separated Hydrogels

### 3.1 Toughness

Toughness is the ability of a material to resist the initiation and growth of cracks under monotonic loading, typically quantified by the fracture energy Γ (J m^−^^2^) using pure‐shear fracture tests. In this set up, a pre‐notched rectangular sample is stretched under uniform deformation to drive steady crack propagation, enabling reliable measurement of intrinsic fracture energy. In some cases, it can also be quantified by the work of extension calculated as the area under the stress–strain curve of un‐notched samples, which reflects the total energy absorbed during deformation prior to failure. In homogeneous elastic networks, Γ is limited by strand scission in a very thin process zone at the crack tip. According to the Lake‐Thomas model, the maximum fracture energy of a single network is proportional to the density of load‐bearing chains, the contour length of the polymer, and the bond dissociation energy [13, 101]. For typical hydrogel networks, with strand lengths on the order of tens of nanometers and crosslink densities of 10^20^–10^21^ m^−3^, this relation yields an upper limit of only 10^1^–10^2^ J m^−2^ [13]. Once covalent bonds at the crack front rupture, the stored elastic energy is released catastrophically, and the crack advances through a nanometer‐scale process zone without any opportunity for stress redistribution or reversible dissipation. Consequently, conventional single‐network hydrogels fail at low fracture energies [14].

In sharp contrast, biological soft tissues exhibit fracture energies that are two to three orders of magnitude higher [14]. Articular cartilage, for instance, withstands cyclic compressive stresses of 4–9 MPa for over one million cycles per year while maintaining a fracture toughness of approximately 10^3^ J m^−^^2^ [102]. This exceptional performance arises from its composite microarchitecture, in which densely packed collagen fibers form a percolating network interpenetrated with proteoglycan macromolecules and hydrated ion‐rich matrices [103]. The collagen network bears tensile stress, while the proteoglycan phase resists compression and supports osmotic pressure. Energy is dissipated through fibril sliding, sacrificial bond rupture, and interstitial fluid migration. Similarly, tendons can achieve tensile strengths exceeding 50 MPa through a hierarchical fibrous organization in which collagen fibrils bundle and align progressively from the nanoscale to the macroscale [104]. This multilevel structure enables sequential recruitment and stiffening of fibril bundles prior to ultimate failure, granting tendons both high mechanical strength and outstanding fracture resistance.

**FIGURE 7:**
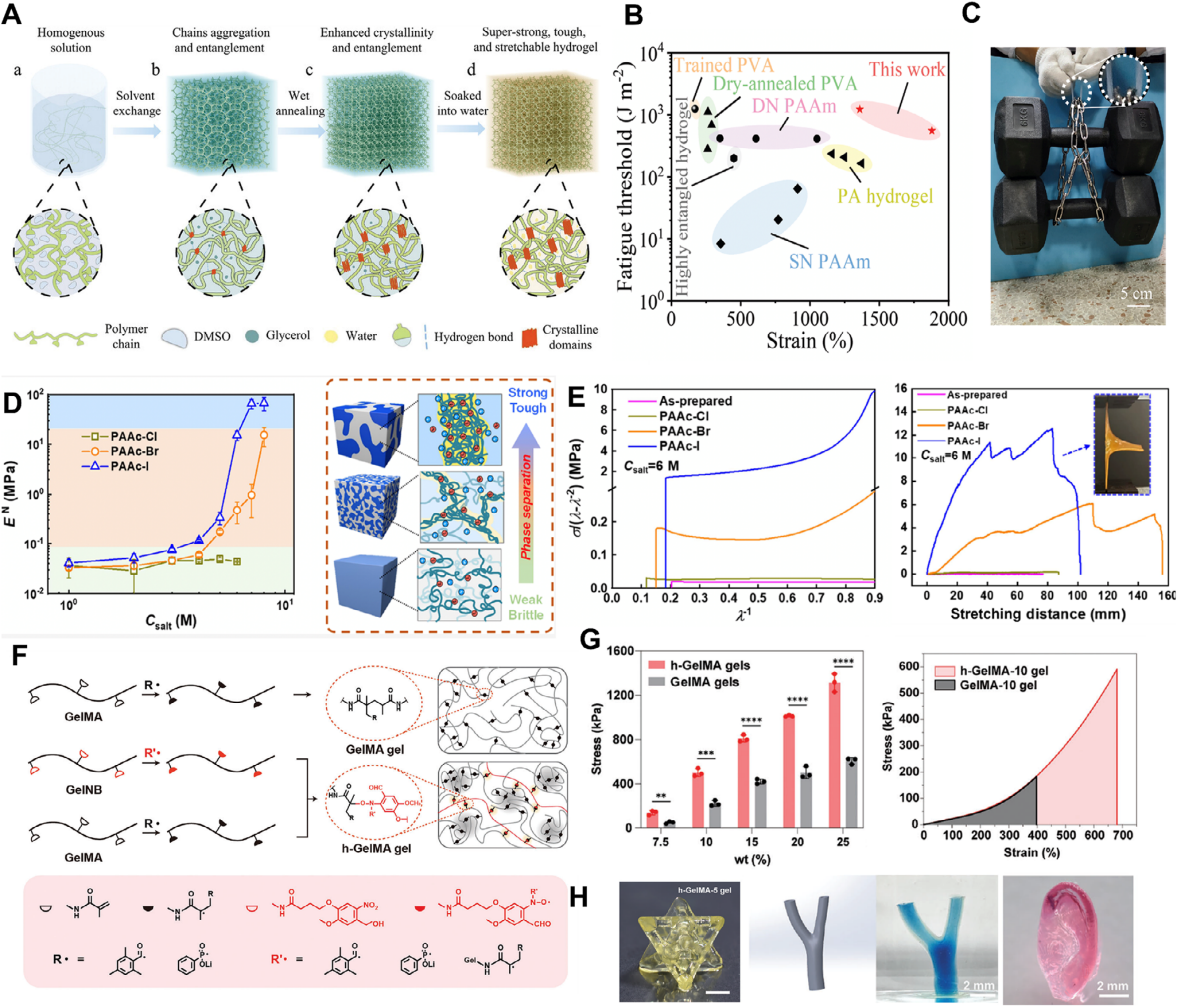
Tough hydrogel achieved by phase separation. (A) Solvent exchange and wet annealing enable the fabrication of PVA hydrogels. (B) The Ashby diagram of fatigue thresholds versus fracture strain among different tough hydrogels. (C) The PVA hydrogel could support 12 kg dumbbells. Reproduced with permission from [107]. Copyright 2023, John Wiley and Sons. (D) Specific counterions induce phase separation in PAA hydrogels and generate dense domains that enhance toughness. (E) Comparison of reduced stress elongation curves for as‐prepared versus ion‐doped PAAc hydrogels [108]. Reproduced with permission. Copyright 2020, American Chemical Society. (F) Schematic illustration of the conventional radical polymerization method and photo‐triggered transient‐radical and persistent‐radical coupling crosslinking mechanism. (G) The tensile strength and fracture strain of the phase‐separated hydrogels (h‐GelMa). (H) 3D bioprinted models with high fidelity for tissue scaffolding [111]. Reproduced with permission. Copyright 2024, John Wiley and Sons.

Early generations of tough hydrogels, particularly those based on double‐network (DN) strategies, demonstrated that the introduction of controlled heterogeneity, i.e., phase separation, can dramatically enhance toughness up to 10^4^ J m^−2^ [105, 106]. Intrinsically heterogeneous DN hydrogels consist of two interpenetrating polymer networks. One is a rigid and brittle, which fractures sacrificially, the other is a soft, ductile second network maintaining structural integrity and redistributing stress. In many polymer‐polymer or polymer‐solvent blends, the structural heterogeneity results from the thermodynamic immiscibility between the components rather than from network interpenetration. It leads to polymer‐rich domains that behave as rigid, load‐bearing regions and solvent‐rich domains that act as compliant matrices [24]. This hierarchical interplay between the rigid and soft phases resembles the stress redistribution mechanism of DN gels, where the soft phase retains network integrity and the stiff phase dissipates fracture energy [23]. One of the recent representative demonstrations of toughness from solvent‐induced phase separation is the solvent exchange‐assisted wet‐annealed poly(vinyl alcohol) (PVA) hydrogel [107]. In this system, Gao et al. combined two sequential solvent exchanges with wet annealing to promote rearrangement of polymer chains (Figure 7A). Glycerol acts as a poor solvent and desolvates polymer chains, forcing the phase separation of PVA into polymer‐rich and polymer‐poor regions. Subsequent wet annealing at 90–120°C enables macromolecular reconstruction and crystallite growth within the polymer‐rich domains, leading to a denser, semi‐crystalline entangled network. The resulting hydrogel exhibits exceptional mechanical properties with a tensile strength of 11.2 ± 0.3 MPa, fracture energy of 25 kJ m^−^^2^, and fatigue threshold of ca. 1.2 kJ m^−^^2^, values that surpass those of the most DN hydrogels (Figure 7B). It could even support two dumbbells (total weight of 12 kg) over 12000 times its own weight (Figure 7C). Alternatively, an amphiphilic entangled hydrogel network was developed by co‐polymerizing hydrophilic poly(ethylene glycol) diacrylate (PEGDA) with hydrophobic poly(*L*‐lactide) (PLA) [68]. Following photopolymerization in dimethylformamide, solvent exchange in water induced phase separation leading to semicrystalline PLA domains tightly woven through the PEGDA network. SAXS/WAXD analyses revealed a domain size of 5–20 nm and partial crystallinity, forming a hierarchical microstructure reminiscent of biological tendon fibrils. The hydrophobic PLA crystallites stiffen the network while the interchain entanglements resist brittle failure, achieving one of the highest modulus‐toughness balances among single‐network hydrogels. A recent study by Zheng and colleagues further demonstrated a phase separation strategy aided by the counterion‐specific effect, achieving extreme mechanical reinforcement in single‐network polyelectrolyte hydrogels [108]. In this work, poly(acrylic acid) (PAA) hydrogels underwent spontaneous phase separation when equilibrated in sodium haloid solutions. Counterintuitively, the larger counterions enhanced hydrophobic polymer‐polymer interactions and accelerated ion‐pair formation between carboxylate groups and Na^+^ counterions in accordance with a reverse Hofmeister series (Cl^−^ < Br^−^ < I^−^) (Figure 7D). This microphase‐separated architecture endowed the hydrogels with extraordinary mechanical performance, achieving fracture stress up to 8.8 ± 1.3 MPa and tearing energy of 29.8 ± 2.4 kJ m^−^^2^, comparable to the state‐of‐the‐art single‐network and DN hydrogels [109] (Figure 7E).

Recent works from Lin and Zhu et al. demonstrated that phase separation can be achieved in situ through a photo‐triggered transient‐radical and persistent‐radical coupling reaction [77, 110, 111]. Specifically, the transient‐radical polymerization of methacrylate components was reinforced by the persistent‐radical coupling with nitroxide species from the photolysis of nitrobenzyl alcohols (NB) in other constituents (Figure 7F) to yield granular nanocomposites. The reaction kinetics creates localized hard domains (∼800 nm in diameter) dispersed throughout the gel matrix, with strong interfacial bonding mediated by the NB coupling. The resulting hydrogels achieve tensile strength up to 1.3 MPa, toughness of ∼1 MJ m^−^^3^, and strain of ∼700%, while maintaining gelatin's degradable and cell‐adhesive properties (Figure 7G). Atomic force microscopy (AFM) modulus mapping confirmed a heterogeneous but cohesive microstructure, where the rigid domains yield plastically and transfer load to the ductile gelatin matrix. Importantly, this approach proceeds in aqueous media and does not require toxic salts making it a biologically safe route to tough hydrogels for 3D bioprinting and tissue scaffolding applications (Figure 7H).

### 3.2 Low Hysteresis

The remarkable fracture energies achieved in phase‐separated hydrogels have revealed the power of structural heterogeneity to toughen soft materials. However, these materials also expose a critical trade‐off between toughness and recoverability (i.e., hysteresis). Hysteresis is characterized by cyclic tensile loading, where the hydrogel samples are repeatedly stretched and unloaded at a fixed strain amplitude to quantify energy dissipation and hysteresis during reversible deformation. Most of the early designs rely on irreversible energy‐dissipating pathways through bond scission, crystalline plasticity, or interfacial debonding, which lead to large hysteresis and cumulative damage under cyclic loading [112, 113, 114]. To overcome this conflict, molecular engineering of the networks, especially the crosslinkers, and mesoscale modulation of high‐order structures that allow elastic deformation energy dissipation have been proposed [115, 116]. Alternatively, slide‐ring hydrogels with topological structure design and hydrogels with nanoconfinement can also achieve hysteresis‐free properties [117, 118]. Other methods including nanoparticle reinforcement and confinement have also succeeded in producing low hysteresis hydrogels [119]. In the scope of this review, we will focus on phase‐separated hydrogels in which both polymer‐rich and polymer‐poor phases form continuous, elastically deformable networks that remain cohesively bonded during deformation. In such systems, the interfaces can delocalize stress without breaking, and the domains deform cooperatively during loading and unloading. As a result, energy is redistributed, allowing these materials to maintain high fracture energy with minimal hysteresis.

**FIGURE 8:**
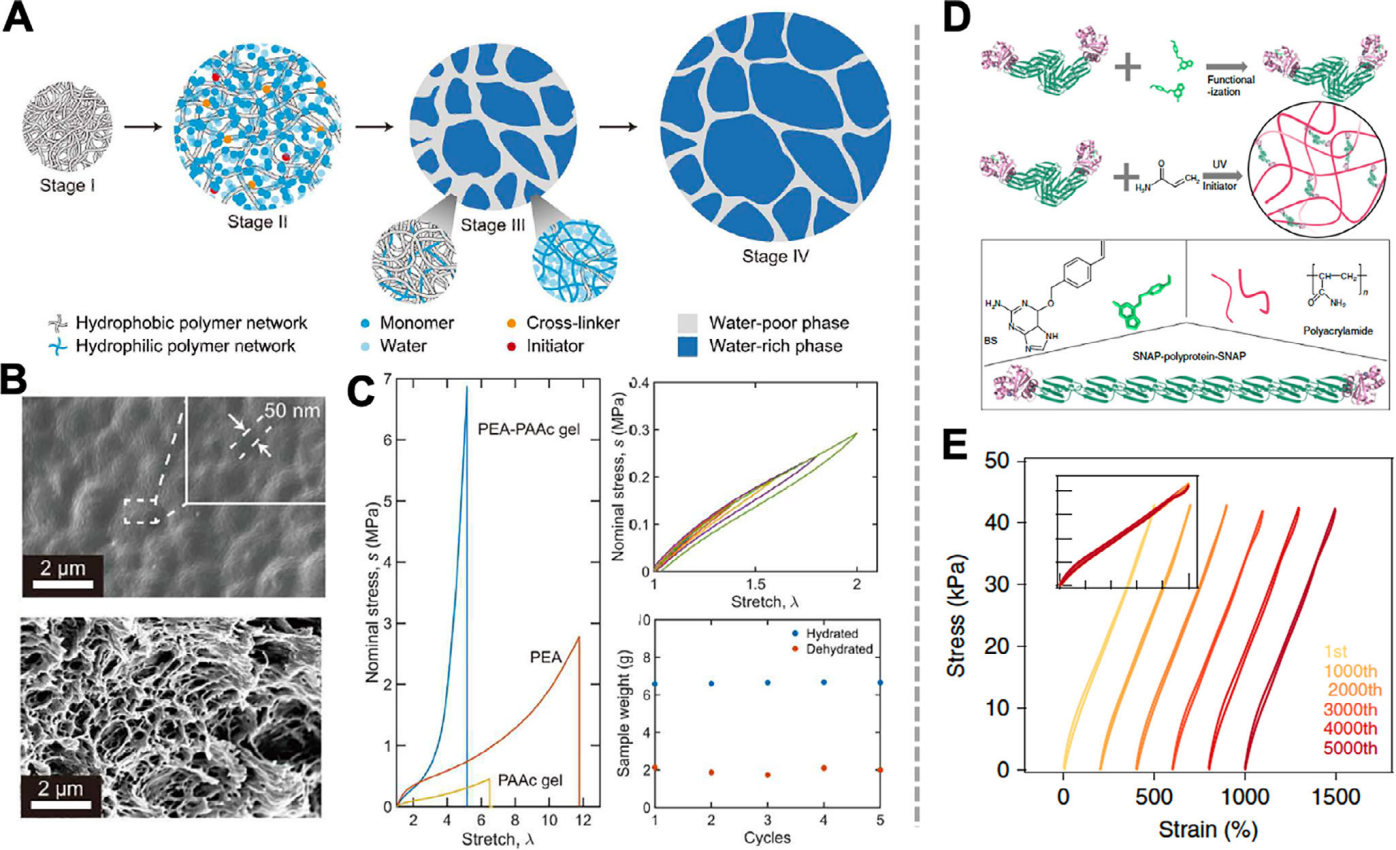
Low hysteresis hydrogel achieved by phase separation. (A) Schematic illustration of the process for preparing PEA‐PAA hydrogel. (B) SEM images of phase‐separated hydrogels with or without swelling. (C) Mechanical tests reveals the superior tensile performance and low hysteresis of PEA‐PAA hydrogel [121]. Reproduced with permission. Copyright 2023, American Association for the Advancement of Science. (D) Schematic illustration of the preparation of the PAA‐G8 hydrogel. (E) The low hysteresis performance of PAA‐G8 hydrogel during 5000 times tensile cycles [122]. Reproduced with permission. Copyright 2020, Springer Nature.

Bicontinuous structures achieve high mechanical efficiency if their interpenetrating networks distribute stress continuously and deform compatibly under load. In a droplet‐type morphology where the droplets are loosely bonded, the local stress mismatch across the weak interfaces often leads to viscous dissipation and thus mechanical failure when strain is applied. In contrast, a bicontinuous microstructure features a topologically interwoven, continuous load‐bearing framework where both rigid (polymer‐rich) and soft (solvent‐rich) domains percolate through the material. Each phase experiences strain proportional to its modulus and volume fraction and the deformation gets partitioned, minimizing local stress buildup and plastic deformation. The reversible elastic deformation eliminates the major microscopic source of hysteresis observed in droplet‐type or crystallite‐reinforced gels. Using nanomechanical mapping and in‐situ SAXS, Gong and colleagues showed that polyampholyte hydrogels spontaneously organize into a multiscale structure composed of hard and soft networks with characteristic length scales between 100 and 500 nm [120]. Specifically, the polyampholyte strands associate through ionic bonds and aggregate into soft and hard regions, forming a bicontinuous network macroscopically. A multiscale energy dissipation mechanism that makes the hydrogel tough and self‐healing was revealed in a combined time‐resolved SAXS and cyclic‐loading analysis: (i) an affine, reversible stage where both networks deform cooperatively and hysteresis remains low, (ii) a non‐affine stage where partial rupture of the hard domains occurs but stress is redistributed through the soft matrix, and (iii) a terminal stage where both networks fail. By leveraging hydrophobicity, Suo and co‐workers introduced the concept of hydrogels of arrested phase separation [121]. Inspired by natural load‐bearing tissues such as tendon, the synthetic analogue was achieved by polymerizing a hydrophilic PAA network within a pre‐formed hydrophobic network of poly(ethyl acrylate) (PEA) (Figure 8A). The chemical disparity between the two polymeric networks promotes demixing into water‐rich (PAA‐rich) and water‐poor (PEA‐rich) phases and the topological entanglements between the interpenetrating networks arrest coarsening at the nanoscale. SEM confirmed a hierarchical morphology with characteristic feature sizes of approximately 50 nm before swelling and about 400 nm after swelling, indicating an arrested spinodal‐type separation that preserves connectivity of both networks (Figure 8B). The resulting hydrogel contained about 76 wt.% water, 19 wt.% PAA, and 5 wt.% PEA, yet it achieved an outstanding toughness‐hysteresis balance with tensile strength of 6.9 MPa, modulus of 0.44 MPa, toughness of 1.7 kJ m^−^^2^, and hysteresis as low as 16.6% under cyclic loading (Figure 8C). However, it should be noted that bicontinuous morphology alone does not necessarily result in low hysteresis. For example, in polyampholyte hydrogels with bicontinuous hard–soft networks, where substantial hysteresis arises from irreversible energy dissipation mechanisms. Gong and co‐workers demonstrated that polyampholyte hydrogels form nanoscale bicontinuous networks of hard and soft domains, yet exhibit large hysteresis during cyclic deformation [120]. Time‐resolved SAXS and recovery tests revealed that, beyond a critical strain, permanent rupture of the hard network occurs while the soft network elastically retracts, leading to significant hysteresis despite full macroscopic shape recovery. A noteworthy analogous example is the polyprotein‐crosslinked polyacrylamide (PAA‐G8) hydrogels which can also achieve low hysteresis through folding and unfolding of the crosslinker within a single network [122]. These hydrogels use vinyl‐terminated tandem‐repeat GB1 polyproteins as covalent crosslinkers within a percolating PAA matrix. During ultraviolet (UV)‐initiated polymerization, the folded proteins form nanodomains dispersed within the flexible polymer network (Figure 8D). The PAA matrix accommodates macroscopic deformation, while the folded GB1 domains remain inactive until local stresses exceed approximately 200 pN, at which point they sequentially unfold and refold after unloading. This mechanism provides nearly complete energy recovery, with hydrogels sustaining 1100% strain and showing less than 5% hysteresis even after 5000 cycles at 500% strain (Figure 8E). The reversible unfolding acts as microscopic “elastic hinges” that dissipate and restore energy without permanent damage, leading to the observed fatigue threshold (∼126 J m^−^^2^).

### 3.3 Fatigue Resistance

While high toughness and low hysteresis govern the performance of hydrogels under single‐cycle deformation, practical applications such as artificial cartilage, soft actuators, and flexible electronics demand mechanical stability over millions of loading‐unloading cycles [81, 123, 124]. Fatigue resistance refers to a material's ability to suppress crack initiation and growth under cyclic loading at stress levels well below its critical fracture strength [125, 126]. In pure‐shear crack‐growth tests, a pre‐existing crack is introduced into a rectangular sample that is subjected to repeated loading cycles under a pure‐shear geometry. The crack extension per cycle is monitored as a function of the applied energy release rate. This approach enables determination of the fatigue threshold, defined as the minimum energy required to sustain crack propagation. The onset of fatigue marks a transition from reversible energy storage to irreversible network damage. Therefore, designing hydrogels with intrinsic resistance to fatigue represents the ultimate pursuit of structural durability [116]. In a homogeneous, covalent network, stress concentrates sharply at the crack tip within a nanometer‐scale process zone, and irreversible bond scission accumulates over cycles. As a result, conventional hydrogels typically display fatigue thresholds Γ_0_ on the order of 10 J m^−^^2^, far below their fracture energies [126]. To enhance durability, modern hydrogel design introduces reversible energy‐dissipative structures that delocalize stress or raise the energy barrier for crack propagation.

**FIGURE 9:**
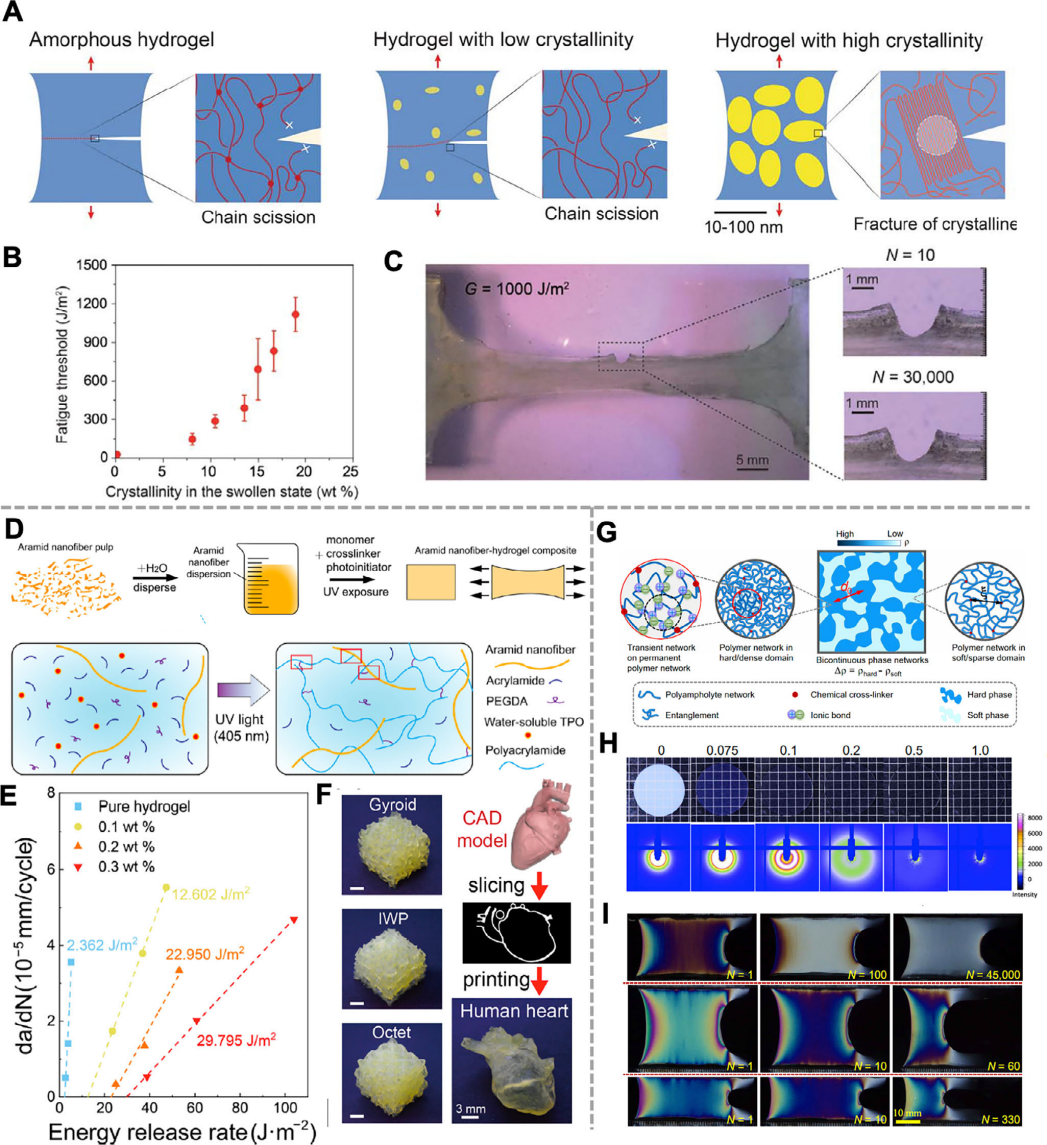
Fatigue resistance of hydrogels enhanced by phase separation. (A) Illustration of fatigue crack propagation in an amorphous hydrogel and in hydrogels with low and high crystallinities under cyclic loads. (B) The fatigue threshold of the hydrogel in the swollen states. (C) Validation of fatigue threshold by using the single notch test [127]. Reproduced with permission. Copyright 2019, American Association for the Advancement of Science. (D) Schematic illustration of the fabrication process of ANF composite hydrogels. (E) Crack growth rate versus energy release rate curves. The fatigue threshold is defined as the x‐axis intercept of the fitted curve representing the crack growth rate versus the energy release rate. (F) Images of 3D printed complex microlattices and human heart using ANF hydrogels [129]. Reproduced with permission. Copyright 2023, Elsevier. (G) The structural illustration of polyampholyte hydrogels. (H) 2D SAXS patterns of phase separation polyampholyte hydrogels with different crosslinker densities. (I) Fatigue testing reveals divergent crack propagation behaviors in strongly *s* weakly phase‐separated gels [82]. Copyright 2021, American Association for the Advancement of Science.

A classic example is the use of crystallization in poly(vinyl alcohol) (PVA) hydrogels, where polymer‐solvent demixing during annealing drives local phase separation followed by chain ordering [127]. As the polymer‐rich regions expel water, they self‐organize into ordered lamellae that coexist with amorphous, hydrated regions, forming a solid‐liquid bicontinuous morphology. The crystalline lamellae serve as permanent elastic crosslinks, while the hydrated matrix provides compliance and distributes stress over a wide amorphous zone around the crack tip (Figure 9A). This synergy raises the energy‐release rate required for crack advance by orders of magnitude, yielding fatigue thresholds exceeding 1000 J m^−^^2^ even at water contents above 80 wt.% (Figure 9B). Notably, no crack extension was observed in the hydrogel samples after 30,000 cycles in the single‐notch test (Figure 9C). The same concept extends to hierarchical composites and conductive networks. In a PVA/silver‐nanowire (AgNW) system, salting‐out drives PVA segregation and crystallite growth while Ag nanowires form a percolating metallic framework [128]. The resulting microstructure consists of a continuous PVA‐rich skeleton interpenetrated by a conductive AgNW network. Under cyclic tension, the PVA crystalline network sustains mechanical load while the metallic phase acts as a crack‐bridging scaffold and retains ultrahigh conductivity. More than 80% stress retention at 3 MPa load was achieved over thousands of cycles, with high conductivity of 958 S cm^−1^.

An analogous example is the aramid‐nanofiber (ANF)‐reinforced hydrogel composite, where hydrophobic ANF nanofibers introduced additional crosslinks and supramolecular interactions as well as promote microphase separation into ANF‐rich and hydrogel‐rich domains during UV‐initiated polymerization (Figure 9D). The formation of ANF‐rich clusters was confirmed by SEM and Fourier‐transform Infrared Spectroscopy (FTIR) spectroscopy, indicative of the demixing of ANFs and the hydrophilic polymer matrix. The resulting nanofiber network effectively anchors polymer chains and redistributes cyclic stress. Even an extremely low ANF loading (0.3 wt.%) in this hydrogel composite was found to be effective in raising the modulus by nearly 30‐fold and increases the fatigue threshold from ∼3 to 30 J m^−^^2^ (Figure 9E) with maintained 3D‐printability and biocompatibility (Figure 9F). In the polyampholyte hydrogels, oppositely charged monomers can undergo polymerization‐induced phase separation, resulting in a bicontinuous network of ionic‐rich and water‐rich phases [81, 82] (Figure 9G). SAXS revealed characteristic domain sizes ranging from 30 to 200 nm, depending on the polymer concentration and charge stoichiometry [82] (Figure 9H). Under cyclic pure‐shear loading, these gels exhibited two regimes of crack propagation, i.e., a slow‐growth regime where cracks advanced less than 0.1 µm per cycle and a rapid‐growth regime once a critical stretch was reached. Polarized light microscopy revealed stress blunting below the transition in strongly phase‐separated hydrogels while persistent butterfly patterns drive rapid crack growth above the transition or in weakly separated networks (Figure 9I). The intrinsic fatigue threshold was measured to be ca. 50 J m^−^^2^, whereas the transition threshold went up to 200–250 J m^−^^2^, indicating that fatigue resistance arises from the energy required to break through bicontinuous domains rather than through single‐chain scission. In situ birefringence mapping demonstrated gradual crack‐tip blunting and stress redistribution over hundreds of micrometers, which is an order of magnitude larger than the process zone of homogeneous gels.

### 3.4 Anisotropy Alignment

Nature provides striking examples of soft materials that combine extraordinary extensibility with dynamic mechanical adaptability. Biological tissues such as tendon, muscle, skin, and cartilage can deform freely at small strains yet stiffen dramatically when stretched, enabling them to bear large loads without rupture [130]. This strain‐stiffening behavior, often accompanied by directional anisotropy, arises from the hierarchical phase organization of their macromolecular constituents. For example, muscle fibers and cartilage exhibit fibrillar or lamellar phase separation between stiff, protein‐rich domains and hydrated, compliant matrices. The cooperative reorientation and engagement of these domains under tension allow tissues to transition smoothly from compliant to stiff states, combining flexibility, toughness, and resilience over repeated cycles.

**FIGURE 10:**
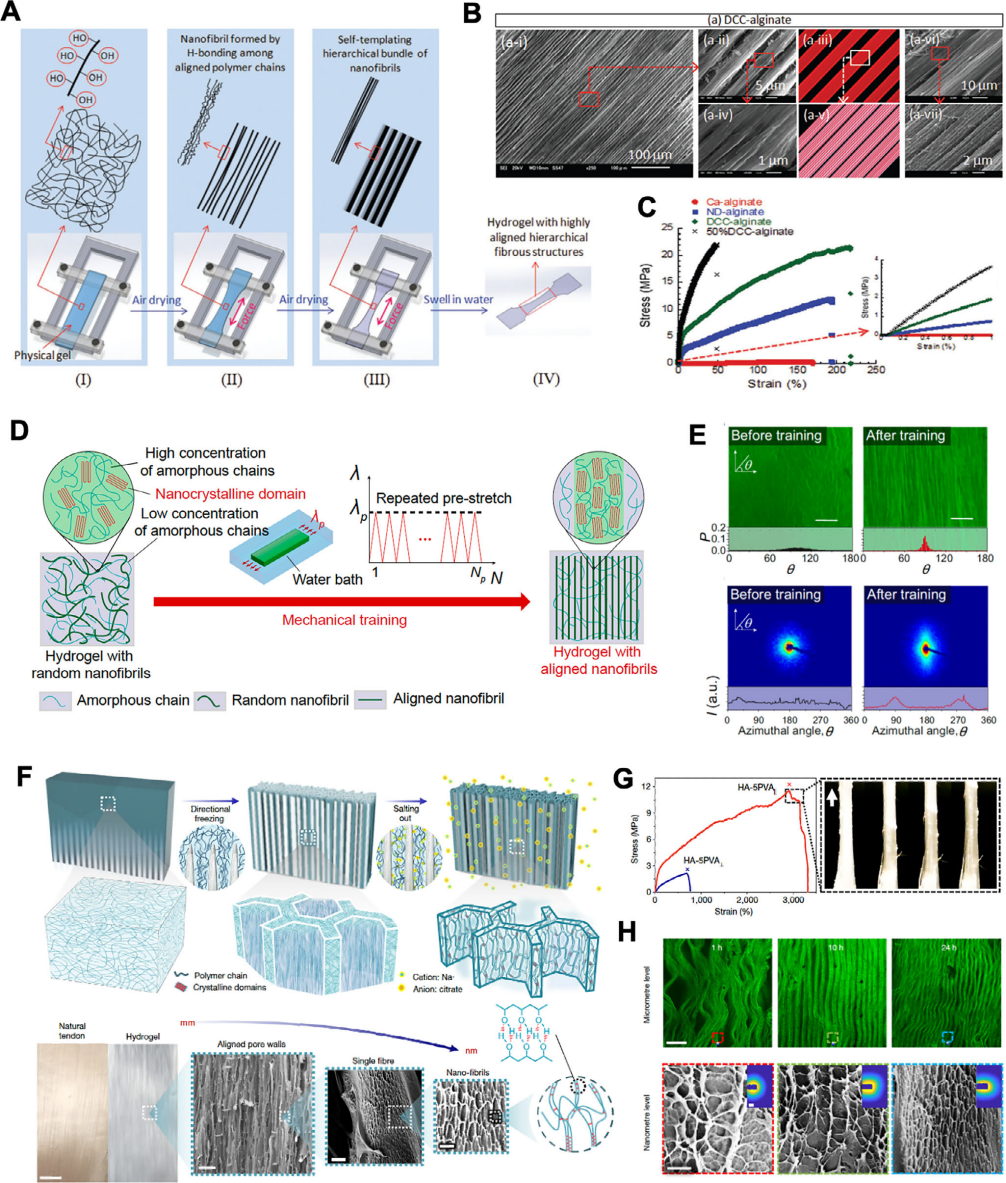
The anisotropy alignment in phase‐separated hydrogel. (A) DCC yields highly aligned fibrous hydrogels characterized by hierarchical superstructures. (B) Structural analysis of DCC alginate gel by SEM imaging. (C) Tensile stress‐strain diagram of alginate hydrogels, with the inset magnifying the low‐strain regime [131]. Reproduced with permission. Copyright 2018, John Wiley and Sons. (D) Mechanical training induces the transition from randomly oriented to highly aligned nanofibrils within the PVA hydrogel. (E) Confocal microscopy and SAXS analysis for PVA hydrogel before and after mechanical training. Reproduced under terms of the CC‐BY license [132]. Copyright 2019, The authors, published by National Academy of Science. (F) Fabrication and hierarchical structures of PVA hydrogels with freeze‐casting and salting‐out processing. (G) Tensile stress‐strain curve and fracture images of the PVA hydrogel. (H) Confocal and SEM images detail the nanofibril network formation [133]. Reproduced with permission. Copyright 2021, Springer Nature.

A bioinspired approach was proposed by Gong and colleagues in 2018, where they developed a drying‐in‐confined‐condition (DCC) process to fabricate anisotropic hydrogels with perfectly aligned hierarchical fibrous structures [131]. Using rigid polysaccharides such as alginate and cellulose, the authors clamped hydrated physical gels at fixed length and allowed slow evaporation of water in air (Figure 10A). The constrained shrinkage imposed uniaxial tension as the gel contracted laterally, aligning polymer chains along the long axis. When the polymer concentration reached about 30 wt.%, polymer‐solvent phase separation occurred, and the chains aggregated into nanofibrils through hydrogen bonding and ionic association (Figure 10B). These nanofibrils further assembled into submicrometer and micrometer‐sized fibers, forming a multilevel fibrous hierarchy that remained stable after reswelling. The aligned Ca‐alginate gel displayed a Young's modulus of approximately 200 MPa, a fracture stress of about 22 MPa, and a work of extension near 31 MJ m^−^^3^ at 57 wt.% water, values comparable to those of natural ligaments (Figure 10C). Alternatively, PVA nanofibrils can repeatedly undergo mechanical training in water to reorient semicrystalline domains (Figure 10D) [132]. SAXS scattering and confocal microscopy revealed that the randomly distributed PVA nanofibrils gradually aligned with the stretching axis after several thousand cycles while maintaining more than 80 wt.% water (Figure 10E). The resulting semicrystalline network with nanofibrils ranging from 100 nm to 1 µm in diameter embedded within an amorphous matrix – similar to muscle myofibrils embedded in sarcoplasm. This mechanically induced alignment produced a fatigue threshold of approximately 1.25 kJ m^−^^2^, a tensile strength of about 5 MPa, and an elastic modulus near 200 kPa, which approach the mechanical range of natural muscle. In situ X‐ray scattering under load confirmed that the crystalline lamellae progressively oriented along the tension direction, leading to a reversible increase in modulus. Crack propagation images further showed that the aligned fibrils pinned and bridged cracks, forcing them to deviate along the fibril‐matrix interfaces. This mechanically trained network demonstrates how stress‐driven rearrangement of semicrystalline domains can mimic the active strain‐stiffening of biological muscle.

A more sophisticated coupling of thermodynamic and structural control was established by He and co‐workers in 2021 through the synergistic freeze‐casting and salting‐out processing [133]. The directional freezing produced micrometer‐scale honeycomb pore walls aligned along the temperature gradient, while the subsequent immersion in a kosmotropic salt solution triggered polymer‐solvent phase separation and crystallization within the walls (Figure 10F). The PVA hydrogels possessed a hierarchical anisotropic architecture comprising nanofibril meshes decorating the aligned pore lamellae, closely resembling that of tendon. These hydrogels retained 70–95 wt.% water yet achieved tensile strengths up to 23 MPa, toughness values of 210 MJ m^−^^3^, and a fatigue threshold of about 10 kJ m^−^^2^ (Figure 10G). Confocal, SEM and SAXS analyses confirmed 40% crystallinity and strong orientation of nanofibrils along the freezing direction (Figure 10H). Under cyclic loading, the nanofibril network reoriented and densified, leading to reversible strain‐stiffening with deflecting cracks at the aligned interfaces. In a subsequent work, a bidirectional ice‐templating method was used to tune the alignment of lamellar pores in PVA hydrogels [134]. By introducing a second temperature gradient through a polydimethylsiloxane wedge, the authors manipulated the growth orientation of ice crystals during freezing, resulting in lamellar architectures with adjustable alignment. SEM and Fourier‐transform analyses showed that the degree of orientation increased with wedge angle up to 40°, leading to a four‐fold increase in modulus and a five‐fold increase in tensile strength along the alignment direction. The anisotropy ratio of moduli between orthogonal directions was tunable between 1.6 and 8.3. A micromechanical model based on Eshelby's equivalence and Mori‐Tanaka mean‐field theory quantitatively reproduced these results, confirming that structural alignment arising from directional phase separation governs the observed anisotropic mechanics.

### 3.5 Responsiveness

Biological tissues not only offer mechanical protection but also continuously sense, adapt, and respond to environmental stimuli like temperature, pH, light, and ionic strength [135, 136]. These abilities originate from the coexistence of multiple dynamic phases in their hierarchical structures. For example, cartilage adjusts its hydration and ion content in response to osmotic pressure, muscle converts chemical gradients into mechanical motion, and skin couples mechanical deformation with electrical signalling [137]. Synthetic hydrogels can also incorporate phase‐separated architectures to evolve from passive mechanical materials into responsive and multifunctional systems capable of sensing, actuation, conductivity, and self‐regulation [138, 139, 140].

**FIGURE 11:**
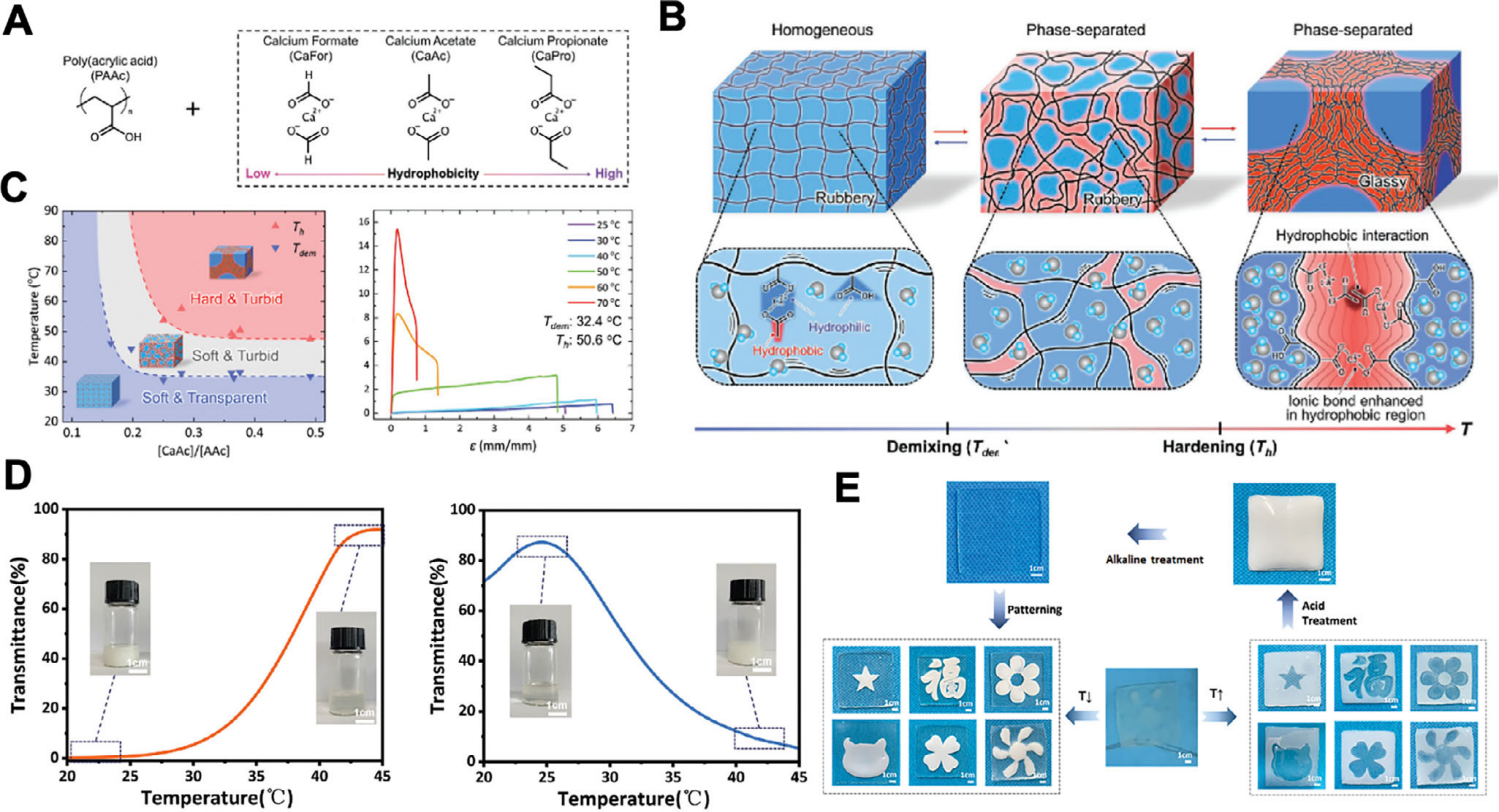
Stimuli responsiveness of hydrogels enabled by phase separation. (A) Molecular structures of calcium acetate PAA hydrogel. (B) Schematic illustration of the mechanism of instant thermal hardening of the synthetic polymer systems. (C) Phase diagram of calcium acetate PAA hydrogels and uniaxial tensile test results at different temperatures [145]. Reproduced with permission. Copyright 2019, Wiley‐VCH GmbH. (D) Temperature dependent transmittance curves characterize the distinct phase transitions of (PACA‐HPC) hydrogels. (E) Thermal‐responsive patterns on (PACA‐HPC) hydrogels [74]. Reproduced with permission. Copyright 2024, Wiley‐VCH GmbH.

Near their miscibility limit, polymer composites undergo reversible demixing into polymer‐rich and solvent‐rich domains in response to small perturbations of temperature, pH, or composition. This process allows hydrogels to couple mechanical, optical, electrical, and adhesive responses to external stimuli. Temperature‐controlled phase separation provides one of the most powerful methods for achieving reversible property switching in hydrogels [141]. LCST systems, such as those based on PNIPAM or PAA‐salt complexes, an elevated temperature breaks polymer‐solvent hydrogen bonds break and strengthens hydrophobic interactions leading to phase separation [74, 142]. Conversely, in UCST systems, such as hydrogen‐bonded copolymers and zwitterionic polyelectrolytes, they become immiscible upon cooling as interchain associations strengthen [143, 144]. A calcium acetate‐PAA hydrogel undergoes LCST‐type spinodal decomposition near 32°C and a glass transition near 50°C (Figure 11A) [145]. The dehydration of acetate ions at elevated temperature reinforced ionic coordination and hydrophobic association, leading to cooperative polymer‐solvent phase separation without volume loss (Figure 11B). This thermohardening process converts a soft hydrogel into a rigid plastic within seconds, resulting in nearly three orders of magnitude and an 80‐fold increase in modulus and strength (Figure 11C), respectively. Cooling reversed the transition and restored the initial elasticity. The rapid, isochoric, and reversible nature of this transformation illustrates how LCST‐driven demixing can enable controlled thermal switching of mechanical states. In a related work, a pH‐gated switch between LCST and UCST behaviors in hydrogen‐bonded poly(acrylic acid‐co‐acrylamide)‐hydroxypropyl‐cellulose (PACA‐HPC) hydrogels was demonstrated [74]. In acidic conditions, hydrogen bonding between carboxylic acids and hydroxyl groups promoted UCST‐type aggregation that dissolves upon heating. Under neutral conditions, deprotonation weakened these interactions and restored LCST behavior (Figure 11D). The reversible transition changes both transparency and mechanical stiffness, enabling thermochromic smart windows and information‐encryption devices (Figure 11E).

### 3.6 Hydrated Adhesion

From the underwater adhesion of mussels and the sealing of bleeding vessels by clots, to the requirement for skin‐mounted electronics amidst perspiration, robust hydrated adhesion emerges as a central property governing both native biological functions and advanced smart materials’ performance [146]. Hydrated adhesion, defined as the ability to form robust bonds at aqueous interfaces like blood‐wetted tissues, remains a fundamental challenge in biomaterials science [147]. The adhesion toughness Γ is commonly expressed as Γ = Γ_i_ + Γ_d_ [20]. Γ_i_ represents the intrinsic interfacial work, determined by the true contact area and specific interactions at the interface. Γ_d_ denotes the bulk dissipation, which originates from inelastic deformation and microstructural energy loss within the hydrogel during crack propagation. For homogeneous hydrogels, interfacial water layers impede intimate contact and disfavor molecular interactions (i.e., low Γ_i_) while the highly solvated polymer network offers few mechanisms for energy dissipation (i.e., low Γ_d_) [147, 148]. In peel tests, an adhered hydrogel layer is detached from a substrate at a controlled angle and peeling rate. The steady‐state peeling force is recorded and converted into an adhesion toughness value. This method provides a quantitative measure of interfacial adhesion strength under well‐defined loading conditions. To achieve sufficient bio‐adhesion, biological systems use specific chemistries (e.g., catechols and plasma proteins) to manage interfacial water and enhance Γ_i_, followed by rapid formation of a solvent‐lean, percolating network (e.g., fibrins) to provide substantial Γ_d_ [149, 150, 151]. Conventional biomaterials approaches to strengthening hydrated adhesion have emphasized on surface chemistry (primers, catechols, aldehydes, ionic bridging) or a covalent bonding design with *N*‐hydroxysuccinimide (NHS) chemistry that couples a compliant interlayer to a tough bulk network [152, 153]. Mimicking the natural design, phase separation offers a broadly applicable route to efficient hydrated adhesion. Phase separation processes like solvent exchange or coacervation densify the polymers at the contact displacing water to increase Γ_i_. The formation of an arrested, solventless percolating skeleton creates sacrificial domains and crack deflection pathways that further amplify Γ_d_ while resisting swelling‐induced failure.

**FIGURE 12:**
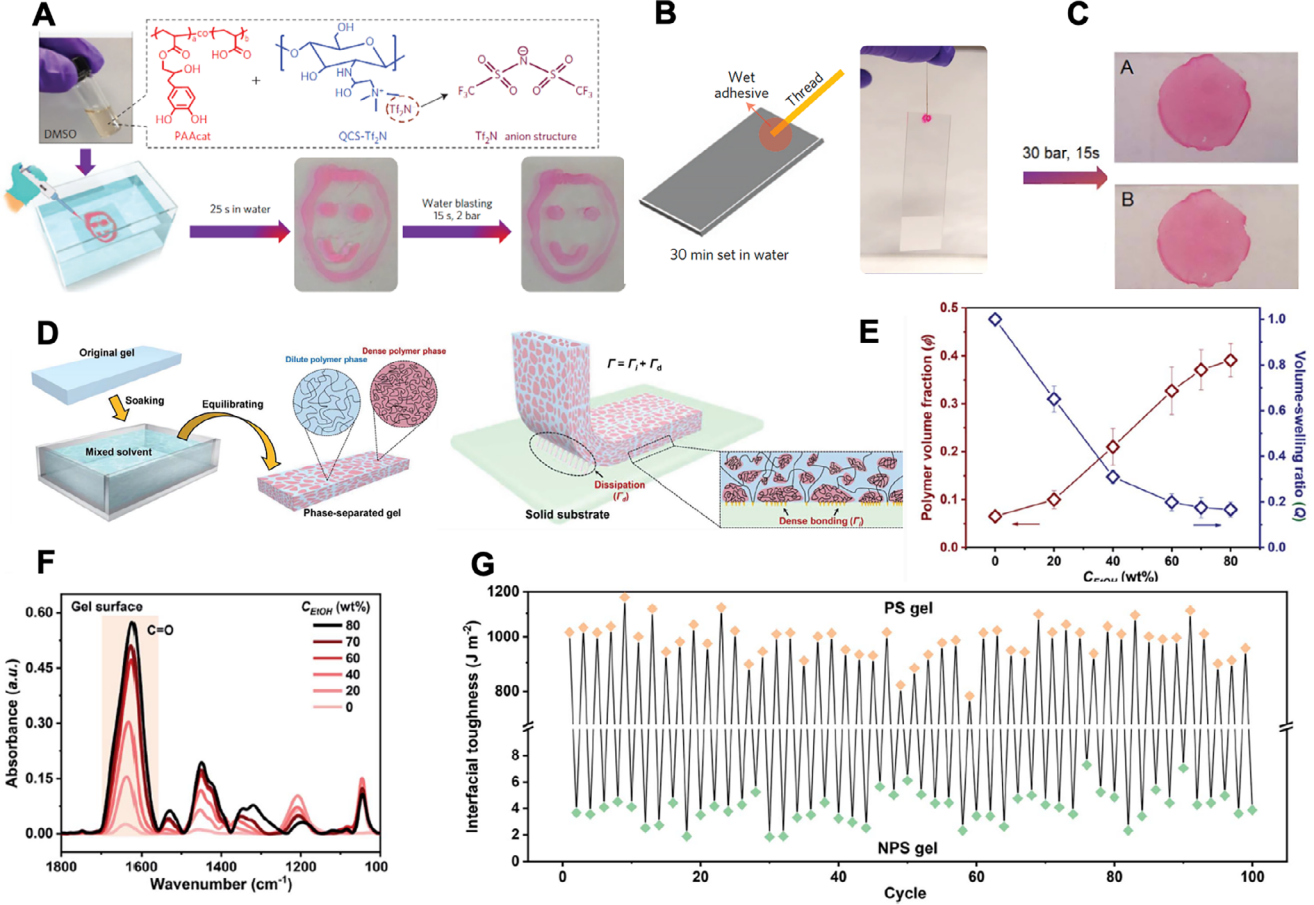
Hydrated adhesion of hydrogels enabled by phase separation. (A) The molecular structure of hydrogel precursor and wet adhesion process assisted by solvent exchange. (B) Casting the polymer blend in water induces robust adhesion to the glass slide, enabling the embedded cotton thread to support the slide's weight. (C) Wet adhesive hydrogel resists higher pressure (30 bar) water blasting [154]. Reproduced with permission. Copyright 2016, Springer Nature. (D) Schematic illustration of the phase‐separation strategy achieving tough adhesion of brittle hydrogels to solid surfaces. (E) Polymer volume fraction and swelling ratio vary as a function of ethanol concentration. (F) FTIR signal intensities of PAAm gels vary with ethanol concentration. (G) Cyclic underwater adhesion performance [155]. Reproduced with permission. Copyright 2021, Royal Society of Chemistry.

A representative example is the solvent exchange‐driven polyelectrolyte complex adhesive. A DMSO solution of catechol‐functionalized PAA and quaternized chitosan underwent water‐DMSO exchange when extruded into aqueous medium, triggering deprotonation, complex coacervation, phase inversion, and porous solidification at the interface (Figure 12A) [154]. The resulting solvent‐lean skin formed rapidly (ca. 25 seconds) on various substrates (i.e., glass slide, Figure 12B) and upon further adhesion development, withstood water‐jet erosion at pressure up to 30 bar (Figure 12C). The robust underwater adhesion stems from the rapid interfacial dehydration and catechol‐mediated interactions which boost the Γ_i_, combined with the porous, percolating microarchitecture that provides a high sacrificial Γ_d_. In a related work, increasing the ratio of the poor solvent (i.e., ethanol) for PAAm hydrogels equilibrated in a water/ethanol mixture induced a controlled phase separation (Figure 12D) [155]. This process caused the gel to deswell significantly and transform into an opaque, heterogeneous network. The structural change drove a dramatic 5.7‐fold increase in the overall polymer volume fraction, from 0.065 in the original gel to 0.371 in the phase‐separated state (Figure 12E). This densification occurred both in the bulk, where it increases network chain density, and at the gel surface, where it increases the concentration of polymer strands. FTIR revealed a surface polymer density that increases with ethanol fraction (i.e., high Γ_i_), while low‐strain rheology indicated a concurrent rise in network chain density, consistent with enhanced bulk dissipation (i.e., high Γ_d_, Figure 12F). In a 90‐degree peeling test with a stiff backing, Γ jumped from ∼2.2 J m^−^^2^ to ∼1.1 × 10^3^ J m^−^^2^ and remained instant and repeatable over >100 attach/detach cycles (Figure 12G). This performance was observed across different solvent pairs and gel chemistries without additional substrate functionalization.

A novel, data‐driven route to hydrated adhesion was recently demonstrated using *de novo* designed statistical‐copolymer hydrogels by Gong and colleagues [156]. These hydrogels were prepared by free‐radical polymerization in DMSO from a monomer library of six amino acid acrylates, selected from adhesive protein databases through a combined data mining and machine learning approach. Following solvent exchange in saline, the formed hydrogels turn opaque, stiffen, and contract slightly. This solvent exchange induces associative clustering into solvent‐lean domains, creating a microphase‐separated structure that functions as a percolating, dissipative skeleton. The top‐performing hydrogel candidates exhibit high underwater tack stresses (exceeding ∼1 MPa on glass) with robust performance across diverse substrates and stable adhesion over repeated attach/detach cycles. This system supports the adhesion framework Γ = Γ_i_ + Γ_d_. Here, hydrophobic/aryl enrichment combined with an optimal level of cationic content promotes interfacial water displacement and specific surface interactions, elevating Γ_i_. Meanwhile, the clustered, solvent‐lean network provides sacrificial dissipation and crack deflection, raising Γ_d_. This was further confirmed by different machine learning model, revealing strong positive contributions from hydrophobic and aromatic monomers and a mid‐range optimum for the cationic fraction, thereby rationalizing the simultaneous gains in both interfacial and bulk adhesion.

### 3.7 Limitations of Phase‐Separated Hydrogels

Despite their demonstrated advantages in mechanical performance, adaptability, and multifunctionality, phase‐separated hydrogels also face intrinsic limitations that must be carefully considered for long‐term and practical applications. For example, most phase‐separated hydrogel architectures rely on kinetically arrested, non‐equilibrated structures, which may evolve over time through domain coarsening, solvent redistribution, or interfacial reorganization. Such structural evolution can lead to gradual changes in mechanical properties, potentially compromising dimensional stability and performance predictability [157]. In addition, under repeated or cyclic loading, stress concentration at phase boundaries can accelerate interfacial fatigue, microstructural damage, or irreversible rupture of load‐bearing domains, particularly when the phases exhibit mechanical mismatch or weak interfacial coupling [82]. In some systems, large energy dissipation is accompanied by permanent network damage, resulting in mechanical hysteresis, softening, or delayed recovery during extended use. Environmental robustness is also a critical concern for phase‐separated hydrogels. Variations in temperature, hydration state, ionic strength, or solvent composition can alter phase stability and reorganization kinetics, leading to unintended morphological changes or functional drift [158, 159]. For example, dehydration–rehydration cycles may amplify phase contrast or induce irreversible aggregation, while changes in ionic conditions can disrupt electrostatic interactions that stabilize phase‐separated domains. In this regard, addressing these limitations requires strategies that balance hydrogel functionality with structural stability. Approaches such as reinforcing interfacial cohesion, introducing hierarchical or covalently anchored phase boundaries, kinetically locking metastable structures, or designing adaptive self‐regulation mechanisms may help improve long‐term reliability and environmental tolerance.

## 4 Applications of Phase‐Separated Hydrogels

Phase separation transforms homogeneous hydrogels into structured soft materials, resulting in coexisting polymer‐rich skeletons and solvent‐rich channels. This biphasic morphology integrates load bearing, dissipative, transport, and interfacial control functions [15, 160, 161]. By programming the processes of demixing and its kinetic arrest, phase separation overcomes entrenched trade‐offs between properties like strength, compliance, adhesion, and conductivity. This capability unlocks performance regimes inaccessible to uniform networks, enabling novel device and therapeutic applications [162, 163, 164]. This section details these applications across biomedical and engineering domains. We will highlight how phase separation serves as a key mechanistic tool for controlling the structure‐property‐function paradigm, thereby addressing specific technological challenges.

### 4.1 Biomedical Applications

Hydrogels have long been recognized as promising materials for biomedical applications, ranging from wound dressings and drug delivery systems to tissue scaffolds and bioelectronic interfaces, owing to their high‐water content, flexibility, and biocompatibility. However, the performance of conventional homogeneous hydrogels is often impeded by their mechanical and interfacial failures in dynamic physiological environments [161]. Phase separation strategies provide a direct route to heterogeneous, hierarchical architectures that mimic the complex structure of native tissues, thereby overcoming these limitations [162, 165]. Additionally, in bioelectronics, phase‐separated hydrogels create bicontinuous pathways that decouple softness from conductivity, enabling stable tissue contact with low impedance and improved resistance to motion artefacts and dehydration [95, 166, 167]. Overall, phase separation provides a universal strategy to integrate mechanical reliability and biological performance, paving the way for advanced hydrogel systems in biomedical applications [20]. In this section, we will discuss several of the latest and most representative applications, including bioadhesives and hemostasis, biosensing and bioelectronics as well as tissue engineering.

#### 4.1.1 Bioadhesives and Hemostasis

Achieving rapid hemostasis on blood‐wetted, deformable tissues is intrinsically demanding. Bonds must form within seconds, withstand physiological pressures, and combine strong adhesion with minimal swelling to prevent seal failure and non‐specific adhesions [147, 168]. Conventional sealants, such as fibrin glues and cyanoacrylates, are constrained by slow setting times, brittle failure, and tissue irritation [169]. Phase separation addresses these limitations by raising the total adhesion toughness Γ through both of its components: Γ_i_ and Γ_d_. Advanced designs further utilize patterned anisotropic/Janus skins to enable one‐sided wet adhesion with an anti‐adhesive opposite face [167].

**FIGURE 13:**
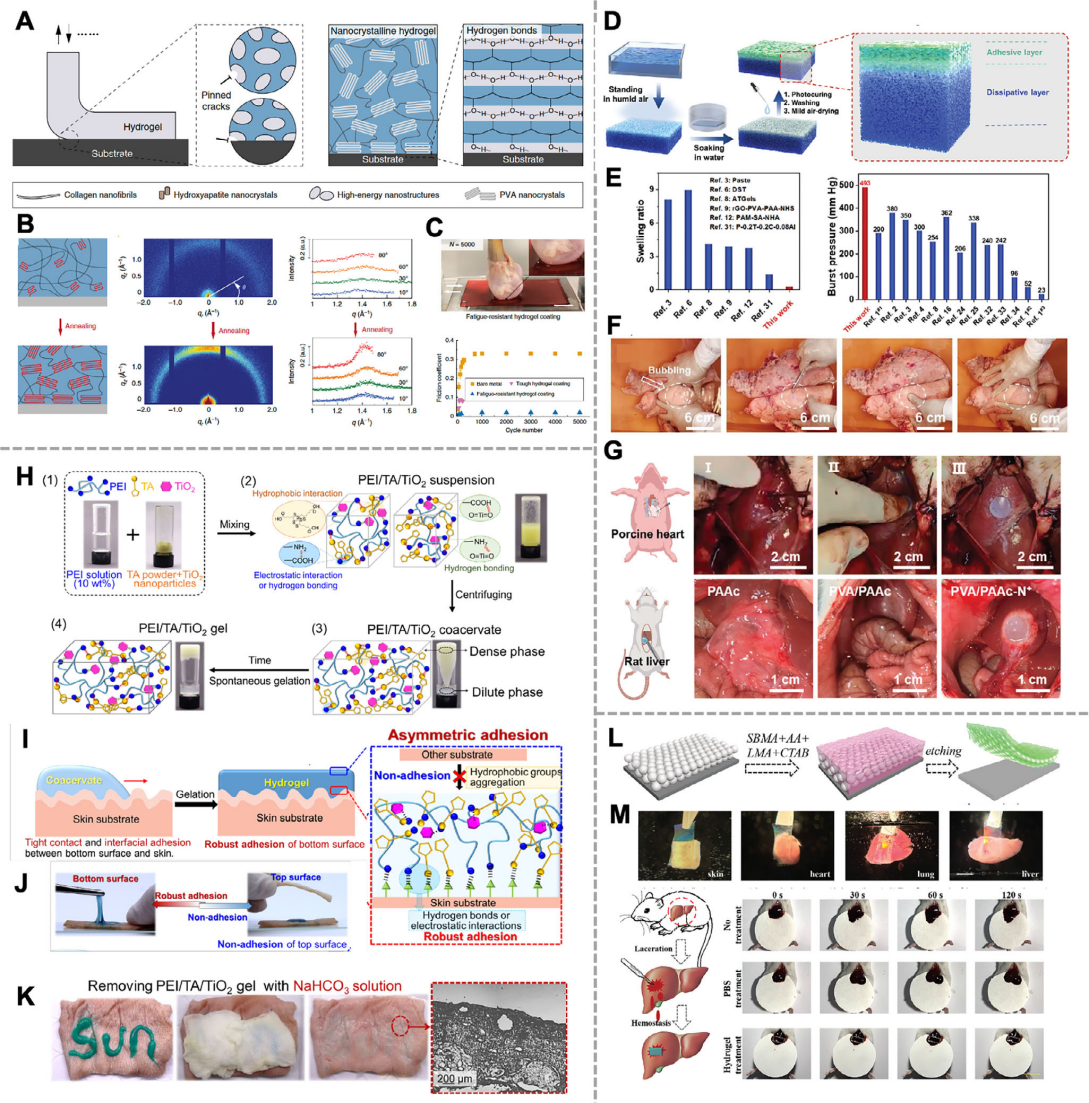
Phase‐separated hydrogel for bioadhesives and hemostasis. (A) Schematic illustration of the fatigue resistant adhesion of PVA hydrogel to substrates. (B) GIWAXS test for analyzing the mechanisms of fatigue resistant hydrogel adhesion. (C) The fatigue resistant hydrogel maintains robust adhesion to stainless steel after 5000 sliding cycles against cartilage. Reproduced under terms of the CC‐BY license [171]. Copyright 2020, The Authors, published by Springer Nature. (D) The phase separation process and structure of PVA/PAA‐N^+^ hydrogel. (E) The swelling ratio and burst pressure tolerance test of PVA/PAA‐N^+^ hydrogel. Ex vivo sealing performance on dissected porcine lung (F) and in vivo sealing and adhesion of ruptured porcine heart and rat liver (G) of PVA/PAA‐N^+^ hydrogels [162]. Copyright 2023, John Wiley and Sons. (H) Schematic illustration for PEI/TA/TiO_2_ hydrogel fabrication. Mechanism illustration (I) and experimental demonstrations (J) of asymmetric adhesion. (K) The removal of PEI/TA/TiO_2_ hydrogel with aqueous NaHCO_3_ treatment on the porcine skin. Reproduced under terms of the CC‐BY license [172]. Copyright 2023, The Authors, published by Elsevier. (L) Schematic diagram of the Janus design and fabrication process of anisotropic adhesion hydrogel. (M) Multi‐organs adhesion and in vivo mice bleeding liver hemostasis property [167]. Reproduced with permission. Copyright 2024, John Wiley and Sons.

A recent study demonstrated that introducing multidentate H‐bond donors (2‐vinyl‐4,6‐diaminotriazine) and deliberately invoking solvent‐induced gel phase separation in a polyion‐complex (PIC) network transforms a typically low‐adhesion PIC into a strong, tunable adhesive [170]. Adhesion strength reached 174 kPa while high tensile strength around 1.2‐2.7 MPa and better extensibility (up to 450% strain) were maintained, with further modulation possible via Hofmeister salts. This design proved that phase‐separated, polymer‐dense skins elevate the interfacial term Γ_i_ via increased true contact and H‐bond/ionic pairing, while the percolated, dissipative skeleton elevates Γ_d_. Zhao et al. realized fatigue‐resistant hydrogel‐solid adhesion via crystallization driven phase separation of PVA at multiple interfaces [171]. The process involves oxygen‐plasma treating substrates (e.g., glass, ceramics, Ti, Al, steel, PU, PDMS), dip‐coating them in 10 wt.% PVA, humidity‐draining (∼1 h), and freeze‐thawing to form a low‐crystallinity gel (Figure 13A). A subsequent dry‐anneal step induced ordered nanocrystalline domains while simultaneously creating dense interfacial H‐bond anchorage to the solid surface. After rehydration, this yielded a thin (∼20 µm), conformal hydrogel coating on complex geometries (optical fibers, springs, ball‐and‐socket joints). Synchrotron‐based grazing incident wide angle X‐ray scattering (GIWAXS) confirmed this annealing‐induced ordering, evidencing PVA chain segregation into nanocrystallites (Figure 13B). Under cyclic 90° peel, the coating showed an interfacial fatigue threshold of Γ_0_ ≈ 800 J m^−^^2^ on diverse substrates and exhibited no interfacial crack advance over 30,000 cycles at G = 800 J m^−^^2^ in water. Γ and Γ_0_ remained stable after 90 days of soaking. Mechanistically, the phase separation‐generated nanocrystals raise Γ_i_ by stabilizing the interface via dense, multivalent H‐bonding to the substrate. They also elevated Γ_d_ by forcing cracks to pull polymer chains out of nanocrystals or along nanocrystal‐substrate contacts, which requires far higher strain energy than fracturing amorphous chains (molecule modeling estimates: ∼50 000–70 000 kJ mol^−^^1^ versus ∼6000 kJ mol^−^^1^). Functionally, the coating sustained low friction and wear against natural cartilage over 5,000 reciprocating cycles, whereas bare metal rapidly degraded (Figure 13C). Complementarily, Ma et al. constructed a moisture‐programmed bilayer hydrogel where both layers are defined by phase‐separation steps [162]. First, PVA in DMSO underwent humidity‐triggered, moisture‐induced phase separation and gelation. Subsequent DMSO‐H_2_O exchange yielded a hydrogel with a porous, interlocking topography and a crystalline, solvent‐lean PVA skeleton, which serves as the dissipative base (Figure 13D). An adhesive prepolymer (PAA‐N^+^) was then infiltrated onto this porous face and UV‐cured in situ to form a wet‐adhesive skin, mechanically keyed into the PVA (PVA/PAA‐N^+^ hydrogel). Tuning the quaternary‐ammonium content (2 wt.%) suppressed swelling via ionic pairing while preserving strength. The resulting photocured phase‐separated bilayer coupled ultralow swelling ratio (0.29) with high shear strength (63 kPa), interfacial toughness (303 J m^−^^2^), and burst‐pressure tolerance (493 ± 39 mmHg, Figure 13E). Ex vivo (Figure 13F) and in vivo (Figure 13G) evaluations demonstrate that the hydrogel withstands dynamic physiological loads to effectively seal heart, intestine, and liver injuries while facilitating rapid suture‐free hemostasis and providing long‐term postoperative anti‐adhesion protection.

Clinical sealants must anchor strongly to target wet tissues yet remain non‐adhesive on their outward‐facing side to avoid secondary sticking and permit atraumatic removal. This necessitates the development of Janus adhesion hydrogels. A coacervate‐derived (LLPS) Janus hydrogel (PEI/TA/TiO_2_) demonstrates how phase separation can be engineered to promote tissue‐side adhesion while suppressing it on the other side [172]. In this work, an aqueous polyethyleneimine (PEI) solution was mixed with a thioctic‐acid/TiO_2_ powder suspension leading to the formation of a fluid complex coacervation (Figure 13H). This fluid phase can rapidly transform into the microtopography of wet skin and then solidify in situ into a cohesive hydrogel. At the tissue interface, anionic motifs (‐COO^−^/‐SO_3_^−^) in the glycocalyx drove counterion exchange with protonated PEI (‐NH_3_^+^) and dehydration of the contact. Simultaneously, hydrogen bonding and carboxylate coordination to TiO_2_ surface sites densified the interface into a polymer‐rich, solvent‐lean skin before the phase separation arrests. At the exposed face, the absence of these polyanionic groups favoured intrabulk PEI‐TA complexation. This triggered oxidation‐assisted disulfide exchange of the 1,2‐dithiolane (thioctic) rings, which immobilized adhesive motifs and enriched the surface with low‐energy dithiolane/TiO_2_ domains (Figure 13I). The result is a charge‐poor, weakly interactive surface with suppressed Γi. In vitro and ex vivo tests confirmed robust, salt‐tolerant adhesion on the tissue‐facing side and weak adhesion on the outer face (asymmetric lap‐shear, Figure 13J). The design also permits reversible detachment in NaHCO_3_, with histology revealing minimal skin damage (Figure 13K). Functionally, the TiO_2_‐laden network demonstrated high durability, maintaining >96% UV‐B shielding after 12 hours in artificial sweat, whereas a commercial sunscreen's efficacy dropped to ∼6.7% due to wash‐off. In vivo studies using a nude‐mouse dorsal‐skin model (311 nm UV‐B, 240 mJ cm^−^^2^ daily for 5 days) confirmed this durable protection. The hydrogel preserved near‐baseline epidermal thickness and keratin levels and suppressed MMP‐9 and IL‐6 upregulation relative to unprotected or commercial‐sunscreen controls. As a complementary Janus design, an emulsion‐templated, inverse‐opal structural‐color hydrogel utilizes phase‐separation‐driven droplet segregation [167]. This process enriches ‐COOH groups at the tissue face while rendering the upper face anti‐adhesive (Figure 13L). The film maintains strong wet adhesion across multiple organs and shortens hemostasis time in a rat liver‐bleed model (Figure 13M). Simultaneously, its structural color and stable ionic pathways provide “seal‐and‐sense” readouts of strain and contact integrity during motion.

#### 4.1.2 Biosensing and Bioelectronics

Flexible electronics are designed to retain full function under deformation, such as bending, folding, and stretching, which offers clear advantages over rigid counterparts in weight, conformability, and on‐body integration [173]. However, a significant challenge arises when these devices must cross the final interface to soft, hydrated, and dynamic living tissue. Biological surfaces are soft with low moduli (ranging from 1 kPa to 10 MPa), high water activity, active ion transport, and constant micromotion. Elastomeric substrates can narrow this mechanical mismatch, but they often lack long‐term biocompatibility and fail to establish stable ionic‐electronic coupling [174]. By contrast, hydrogels are natural candidates for bioelectronic interfaces. They can effectively match tissue mechanics and hydration, facilitate ion transport, and form intimate, conformal contact. Despite these advantages, homogenous gels often fail to provide low and stable contact impedance, maintain high conductivity under large strain, or ensure long‐term durability in saline, sweat, or blood without signal drift or delamination [175].

By reorganizing a homogeneous network into coexisting polymer‐rich and solvent‐rich domains, phase separation effectively decouples the material's softness from its transport and interfacial performance. Critically, phase separation induced solvent‐lean skins can dehydrate and stabilize the tissue contact, while an internal, percolating conductive skeleton transports charge efficiently without sacrificing overall compliance. Among conductive hydrogels, PEDOT:PSS is notable for its high conductivity and biocompatibility [176]. However, its native morphology which consists of a complex of conductive PEDOT with insulating and swell‐prone PSS fundamentally limits both conductivity and stability. Phase separation strategy resolves this intrinsic conflict by reorganizing the material's morphology rather than altering its chemistry. Treatments using non‐solvents, acids, or ion exchange drive phase separation, compelling the PEDOT‐rich domains to self‐assemble [97]. This process promotes π‐π stacking and forms percolating conductive pathways, which dramatically lowers bulk impedance. Simultaneously, this reorganization creates PSS‐lean (or PEDOT‐rich) surface layers. These surfaces are less prone to swelling, which significantly reduces contact impedance and motion artifacts [177]. Crucially, the retained hydrated (solvent‐rich) channels ensure the hydrogel maintains a tissue‐level modulus and essential ionic coupling.

**FIGURE 14:**
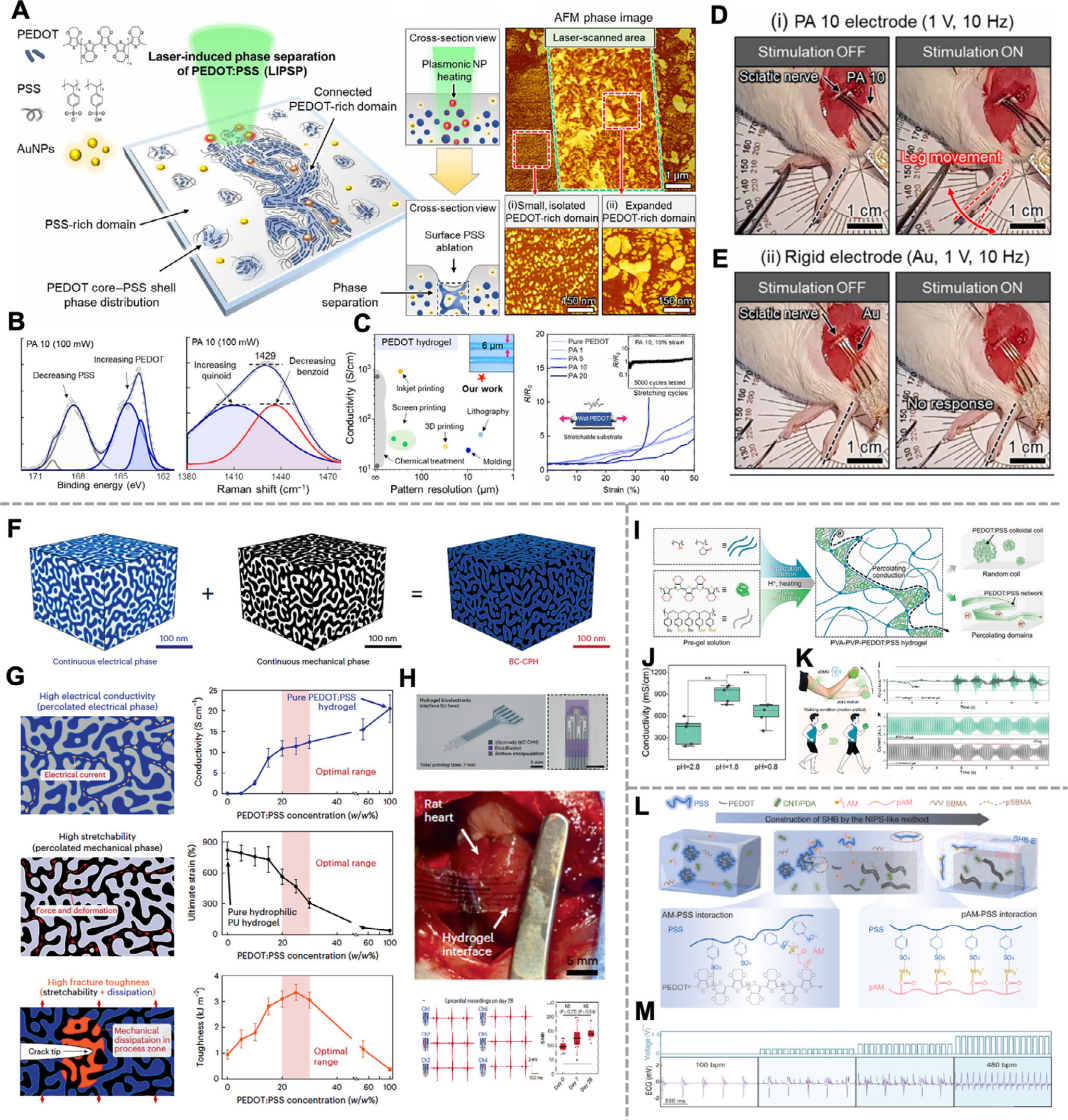
Phase‐separated hydrogel for biosensing and bioelectronics. (A) Schematic illustration of the LIPS hydrogel formation process. (B) XPS and Raman tests of LIPS hydrogel. (C) The resolution and resistance stability in 5000 tensile cycles. (D) LIPS hydrogel‐based electrodes enable high‐fidelity neural recording and signal analysis in the mouse hippocampus. (E) Sciatic nerve stimulation validates the efficacy of LIPS hydrogel electrodes and demonstrates the tunable leg movements modulated by frequency and amplitude. Reproduced under terms of the CC‐BY license [178]. Copyright 2022, The Authors, published by American Association for the Advancement of Science. (F) Illustration of continuous electrical phase, continuous PU phase, and bicontinuous phase hydrogel. (G) Schematic illustration showing how the optimal electrical conductivity, ultimate strain, and fracture toughness are achieved. (H) In vivo electrophysiological recording [179]. Reproduced with permission. Copyright 2023, Springer Nature. (I) Schematic illustration of the preparation of conductive bicontinuous hydrogel for electronics. (J) The conductivity of the bicontinuous hydrogel. (K) The hydrogel‐based motion sensing and signals recording [180]. Reproduced with permission. Copyright 2024, John Wiley and Sons. (L) The fabrication method of hydrogels featuring a PEDOT‐rich core and a PSS‐rich shell. (M) ECG signals of hydrogel at different electrical stimulation voltage [181]. Reproduced with permission. Copyright 2024, Elsevier.

At the high‐resolution end of the processing landscape, laser‐induced phase separation (LIPS) provides a maskless, digital route for fabricating microelectrodes. In this technique, a continuous wave laser (e.g., 532 nm) scanned across an Au‐nanoparticle‐seeded PEDOT:PSS film delivered highly localized photothermal energy and an associated electric field [178]. This energy rapidly drives demixing and conformational reorganization, which is then quenched within milliseconds as the temperature drops (Figure 14A). During this process, PEDOT‐rich domains expand and interconnect, while insulating PSS is diminished or redistributed. This morphological transformation, confirmed by AFM, X‐ray photoelectron spectroscopy (XPS), and Raman spectroscopy, reveals domain coarsening and a characteristic benzoid‐to‐quinoid shift, which is consistent with enhanced carrier density and mobility in an aqueous environment (Figure 14B). After selective washing, the resulting hydrogel, with resolutions as fine as 6 µm, exhibits outstanding properties, including high conductivity (up to 670 S cm^−^^1^ in PBS), sufficient tissue softness (57 MPa), and excellent resistance stability (≥5000 cycles at 15% strain, Figure 14C). This performance translates directly to high‐fidelity neural interfaces. On acute mouse hippocampal slices, patterned microelectrodes conformed to the CA3 region and successfully recorded both single‐unit spikes and state‐dependent local field potentials (LFPs). The fidelity of the hydrogel‐tissue interface is demonstrated by its ability to track pharmacologically‐driven dynamics. Upon perfusion of bicuculline (an excitatory state trigger), the LFP power spectrum correctly shifts to higher frequencies and larger amplitudes, while maintaining a stable, low‐noise baseline. In peripheral nerve applications, cuff‐style LIPS‐hydrogel electrodes wrapped around the sciatic nerve elicited robust motor recruitment (leg kicks, Figure 14D) at significantly lower drive voltages than rigid gold controls (Figure 14E). At 10 Hz, the hydrogel achieved clear recruitment around 0.4 V, much lower than the 2 V required for gold. This work demonstrates that phase separation is critical for improving their performance, where the percolated PEDOT network provides low bulk resistance and the PSS‐lean surface lowers contact impedance, and the hydrated matrix ensures conformal contact.

In parallel with digital fabrication, ink‐level control of demixing enables printable, device‐scale solutions [179]. A key example utilizes a pre‐miscible water/ethanol‐based ink comprising an electrical phase (PEDOT:PSS) and a mechanical phase (a soft polyurethane, PU). During the drying process, the ink was engineered to pass through a spinodal‐like decomposition window, causing both phases to percolate and form a bicontinuous network before the morphology is arrested (Figure 14F). The resulting hydrated material consists of a PEDOT‐rich pathway for charge transport, fully interpenetrated by a solvent‐lean PU skeleton that bears mechanical load and suppresses swelling. This architecture fundamentally breaks the conventional trade‐offs of conductive hydrogels, yielding a material that is simultaneously highly conductive (σ > 10 S cm^−^^1^), exceptionally stretchable (> 400%), tough (fracture toughness in the kJ m^−^^2^ range), and soft (sub‐MPa modulus, Figure 14G). Its impedance remains remarkably strain‐insensitive, as current is confined to the PEDOT network while strain is partitioned to the compliant PU network. This strategy allows for printing entire all‐hydrogel device stacks, including conductors, adhesives, and insulators. The in vivo performance is exceptional, as demonstrated by epicardial arrays on rat hearts that conformed to the moving surface and delivered crisp, multichannel signals from implantation through day 28 (Figure 14H). On the sciatic nerve, cuff‐style interfaces demonstrated extraordinary long‐term stability, with recruitment curves on day 56 nearly overlapping those from day 0, indicating a stable, low‐inflammation interface despite constant motion.

In an acid‐based lock‐and‐stabilize approach, strong acid treatment simultaneously gelates a host polymer (PVA/PVP) while inducing phase separation in the PEDOT:PSS by protonating PSS and screening its Coulombic binding to PEDOT (Figure 14I) [180]. The freed PEDOT‐rich domains expand, densify, and gain π‐π order, while the host locks in a solvent‐lean, elastic framework. The resulting bicontinuous material delivered S cm^−^^1^‐class conductivity (Figure 14J) at tissue‐level moduli (10^2^‐10^3^ kPa), enabling motion‐insensitive skin‐mounted electrodes (Figure 14K). The phase‐separated morphology itself provides this stability by allowing the hydrophilic part to maintain ionic coupling, while the acid‐fixed skeleton blocks swelling and coarsening. A related self‐encapsulation method, driven by a non‐solvent, established a PEDOT‐rich core network while a PSS‐rich outer shell spontaneously formed as a compliant, protective skin (Figure 14L) [181]. The resulting composite was exceptionally soft (10‐20 kPa) and stretchable (>300%), enabling clean in vivo electrocardiogram (ECG) and low‐threshold cardiac pacing (Figure 14M). Taken together, these diverse processing strategies, from digital laser‐patterning to one‐pot chemical fixation, converge on a powerful design logic. To create high‐performance bioelectronic hydrogels, one must: (1) build a percolated PEDOT network to secure low, strain‐robust bulk impedance, and (2) sculpt a solvent‐lean, mechanically supportive phase (e.g., PU, PVA, or a PSS‐rich skin) to stabilize the interface, suppress swelling, and ensure long‐term contact.

#### 4.1.3 Tissue Engineering

Phase separation provides a powerful method to partially decouple and tune a hydrogel's mechanical, transport, and bioactive properties by controlling key parameters like thermodynamic driving forces, arrest kinetics, and crosslink density. A notable application is the generation of perfusable, porous channels for tissue engineering [182, 183]. Among the phase separation routes, LLPS, which includes both segregative ATPS and associative LLPS, is especially bio‐friendly as it mimics the formation of cellular condensates and ECM [15, 93]. This control over the process enables precise architectural programming. Stronger thermodynamic driving forces can yield bicontinuous, cell‐scale porosity while weaker forces result in droplet‐matrix morphologies. The subsequent arrest by covalent crosslinking, crystallization, or associative networking kinetically fixes these architectures. This strategy allows for the creation of scaffolds with highly connected pores for perfusion, reinforced walls for load bearing, time‐programmed maturation for evolving modulus, and intrinsic partitioning to pattern biochemical cues [184]. Furthermore, demixing near the gel point imparts shear‐thinning and rapid recovery, enabling injectable or printable bioinks under all‐aqueous, cell‐compatible conditions [185]. In this section, we will highlight hydrogels derived from either associative LLPS or ATPS for their applications in tissue engineering.

**FIGURE 15:**
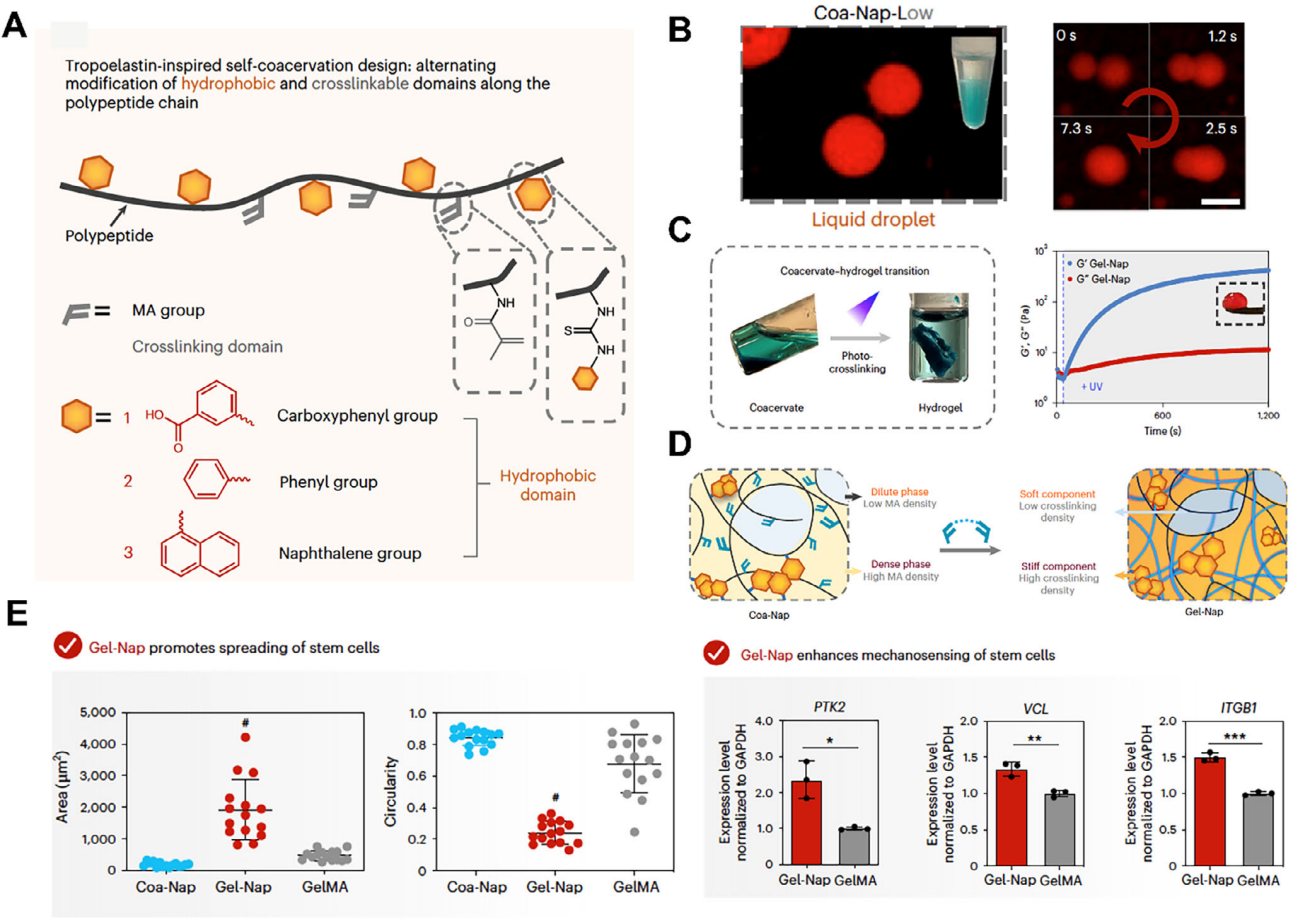
LLPS hydrogel for tissue engineering. (A) Schematic of the tropoelastin‐inspired minimalistic model comprising alternating hydrophobic moieties and covalent crosslinking domains. (B) Fluorescence images of coacervates and droplets coalescence. (C) The image and rheological time‐sweep test show that the covalent crosslinked coacervate transformed into the hydrogel. (D) The coacervate‐hydrogel transition introduces structural heterogeneity into the coacervate‐derived hydrogel. (E) Human MSCs exhibit accelerated spreading, mature focal adhesions, and nuclear YAP translocation, accompanied with the upregulation of adhesion and mechanotransduction genes [165]. Reproduced with permission. Copyright 2025, Springer Nature.

Building directly on the principles of associative LLPS, recent work has leveraged controlled demixing to engineer sophisticated biomimetic architectures with spatially programmed mechanics and porosity. For example, Xie and colleagues employed a minimalist, tropoelastin‐inspired scheme by endowing gelatin with a calibrated density of hydrophobic motifs (naphthyl groups) and a photocrosslinkable fraction (Figure 15A) [165]. Under mild, all‐aqueous conditions, multivalent π‐π and hydrophobic interactions drive genuine liquid–liquid coacervation. The system forms micron‐scale droplets that fuse and mature, confirmed as liquid condensates by their sensitivity to hydrotropes and rapid molecular exchange (Figure 15B). A brief UV exposure then arrests the condensate at a chosen stage, converting the emulsion into a heterogeneous hydrogel by fixing the crosslinkable motifs that have been concentrated within the dense phase (Figure 15C). The resulting material is mechanically heterogeneous, consisting of interspersed stiff and soft domains at the cellular scale (Figure 15D). This mesoscale compartmentalization, rather than the average bulk modulus, was identified as the primary driver for mechanosensing. Human mesenchymal stem cells (hMSCs) cultured on the material displayed faster spreading, mature focal adhesions, and nuclear YAP translocation, with adhesion and mechanotransduction genes upregulated (Figure 15E). Because the entire sequence is cytocompatible, living cells can be embedded directly during coacervation. This enables the mechanical cue duration to be programmed by the timing of arrest without relying on complex multi‐network chemistries. Advancing this concept from physical templating to covalent patterning, the same group developed a system by blending a thermoresponsive macromer with a photocurable gelatin precursor [186]. Upon warming, the system underwent LLPS, causing the thermopolymer to partition into dense droplets within a dilute, gelatin‐rich continuous phase. Photogelation initiated during this demixed state spatially compartmentalizes the crosslinking. The single network yielded by this process is chemically uniform but mechanically heterogeneous. It consists of highly crosslinked, micrometer‐sized domains, which originated from the droplets, embedded within a more compliant matrix. Underwater nanoindentation confirmed a modulus landscape varying between 0.2 and 10 kPa over tens‐of‐micrometre periodicities, matching the length scale of cellular mechanosensing. This stiffness microheterogeneity was shown to causally accelerate osteogenic differentiation through the recruitment of specific pathways, including talin‐mediated microtubule‐actin crosstalk, αTAT1‐dependent microtubule acetylation, and elevated autophagic flux. These compartmentalized networks supported denser, better‐organized in vivo bone regeneration in calvarial defect models compared to homogeneous controls, demonstrating conclusively that the spatial distribution of crosslink density is decisive for regeneration. Exploiting a similar logic for a different purpose, Yasue and colleagues utilized associative LLPS during the Michael‐type gelation of gelatin [187]. Here, quadruple hydrogen bonding (UPy‐UPy) moieties drive the condensation of a transient dense phase concurrently with covalent network formation in the dilute phase. After gelation, the selective dissolution of this UPy‐rich sacrificial fraction opens an interconnected, capillary‐like microporosity (single‐digit to tens of micrometres) throughout the soft, load‐sharing skeleton. This architecture uniquely reconciles injectability with rapid mass transport; the pores facilitate MSC spreading, proliferation, and paracrine signalling, while the elastic scaffold maintains mechanical integrity. In volumetric muscle‐loss models, these LLPS‐templated gels improved graft survival and host myoblast infiltration, leading to superior functional recovery. This work provides a powerful demonstration of using demixing to engineer distinct transport pathways and load‐bearing phases in situ for immediate translational benefit.

**FIGURE 16:**
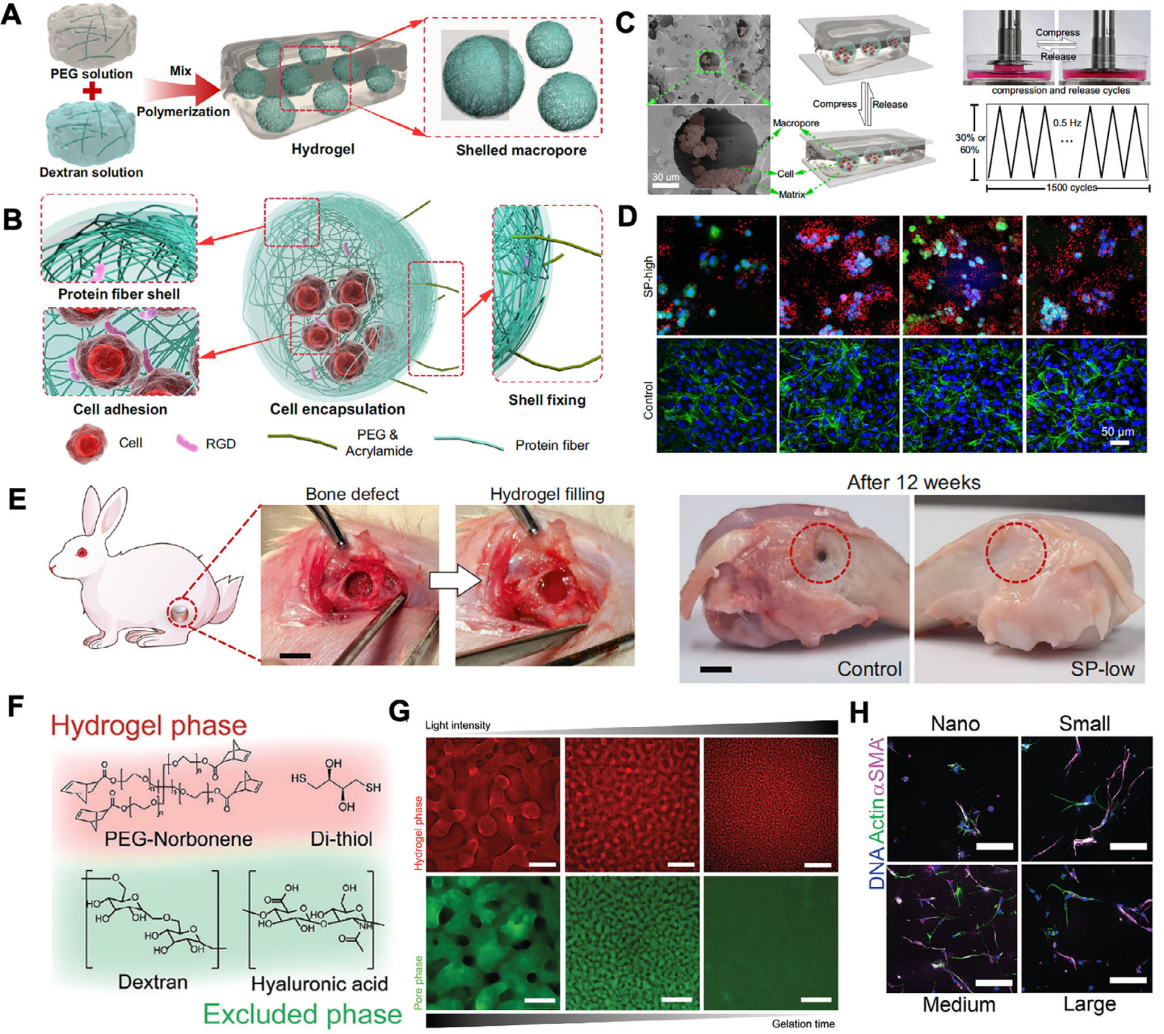
ATPS and PIPS hydrogels for tissue engineering. (A) Schematic represents the fabrication and structure of microporous hydrogel. (B) Schematic illustration depicts the shell‐hardened macropore structure and cell encapsulation. (C) The real‐time mechanical monitoring demonstrates the effective protection of encapsulated cells within shell‐hardened macropores during continuous compression‐relaxation cycles. (D) Cell proliferation and osteogenic differentiation are promoted by shell‐hardened microporosity. (E) Bone regeneration using the rBMSC‐encapsulated hydrogel in a New Zealand white rabbit model [94]. Reproduced with permission. Copyright 2025, Springer Nature. (F) The molecular components of precursor solution for the macroporous system. (G) Fluorescence imaging distinguishes the gel phase (red) from pores (green) in hydrogels fabricated under different light intensities. (H) Fluorescence images of cells in the nanoporous and macroporous hydrogels with different pore sizes [77]. Reproduced with permission. Copyright 2025, John Wiley and Sons.

Strategies based on ATPS demonstrate how demixing can be harnessed to couple cell‐scale structure with regenerative function. For example, Chen and colleagues utilized a PEG/dextran aqueous two‐phase emulsion as a one‐step platform to build mechanically contrasted yet covalently integrated hydrogels [163]. By crosslinking both phases while still in the liquid emulsion state, this method yielded a covalently interpenetrated interphase, with interfacial mechanics markedly stronger than those from sequential assembly. This platform allows for the orthogonal programming of mechanics by exploiting selective partitioning and phase‐specific post‐processing. For instance, alginate added exclusively to the dextran‐rich phase creates a dissipative DN subnetwork (via Ca^2^^+^‐alginate) that selectively stiffens that domain. Alternatively, substituting the PEG phase with PVA and applying salting‐out processing facilitate H‐bonding and crystallite formation, resulting in a tendon‐like modulus (E_PVA_ ≈ 3.0 ± 0.1 MPa). The opposing dextran phase remained ultra‐soft and highly extensible (E_DEX_ ≈ 3.4 ± 1.2 kPa; λc ≈ 900%). The resulting hydrogel is covalently knitted and fabricated in a single demix‐and‐cure step. A complementary advance pushes ATPS templating beyond creating simple bulk contrast, enabling the engineering of macroporous architectures with reinforced boundaries. Xue and co‐workers induced a PEG/dextran liquid–liquid separation where PEG forms the polymerizable, percolating phase, and dextran forms non‐percolating droplets that serve as macropore templates (Figure 16A) [94]. Critically, lysozyme nanofibers were simultaneously self‐assembled at the PEG/dextran interfaces and covalently acryl‐anchored during polymerization (Figure 16B). This process creates rigid, proteinaceous pore shells decorated with RGD. This design effectively decouples matrix degradability from shell rigidity. The bulk matrix can be tuned to degrade gradually, while the persistent shells furnish sustained mechanical cues and shield encapsulated stem cells from compressive forces (Figure 16C). This shell‐hardened macroporosity enabled rapid cell proliferation and osteogenic differentiation in vitro (Figure 16D). The materials drove significant bone repair in vivo in both rabbit and porcine femoral condyle defect models, improving bone volume and trabecular architecture (Figure 16E). ATPS templates the pore topology, interfacial protein assembly creates a stiff shell that outlasts the degrading matrix, and covalent anchoring arrests the entire structure, thereby providing continuous mechanical signalling that uniformly degradable gels cannot maintain.

This design principle also extends beyond all‐aqueous partitioning to photopolymerization‐induced phase separation (PIPS), which offers rapid, in situ spatiotemporal control. In this approach, an initially miscible resin (e.g., in thiol‐ene and PEG/dextran systems) is driven across the binodal by light (Figure 16F) [77, 96]. As polymerization increases molecular weight, it reduces mixing entropy and initiates demixing that is then kinetically arrested by the stiffening network. This process yielded ECM‐like scaffolds with interconnected pores (ranging from 2 to 200µm) whose topology is precisely tunable by light intensity, composition, and irradiation rates (Figure 16G). Functionally, these PIPS‐generated architectures accelerated osteogenesis and fibroblast migration (Figure 16H). The results show that demixing‐set permeability, rather than average bulk modulus, is a primary determinant of early cellular behavior. Furthermore, the ability to volumetrically print these porous, cell‐laden constructs in seconds highlights the power of PIPS to hard‐wire perfusion and guidance cues into scaffolds on demand [84]. The common goal of these scaffold designs is to use a phase‐separation route that reproducibly generates a percolating macropore network (for mass transport) that remains chemically and mechanically integrated with a continuous, load‐sharing matrix (for robust mechanics). This concept provides a practical bridge from the physics of processing parameters to the biology of predictable regenerative function.

### 4.2 Mechanical and Engineering Applications

In parallel with their impact in the biomedical field, phase‐separated hydrogels are emerging as foundational materials for mechanical systems like soft robotics and flexible device interfaces [98, 113, 128]. These applications demand a unique fusion of tissue‐like compliance with high fatigue tolerance and stable conductivity which conventional homogeneous gels cannot achieve. Phase separation overcomes this limitation by creating percolated skeletons and segregated conductive pathways that decouple the material's mechanical and electronic responses [188, 189]. Accordingly, the following sections will detail these innovations, first in soft robotics and actuators by examining anisotropy and morphing enabled by phase separation, and subsequently in wearable and flexible devices, highlighting interfaces and signal fidelity stabilized by phase separation.

#### 4.2.1 Soft Robotics and Actuators

Soft robotics, a field inspired by the adaptive and compliant nature of biological systems, presents a paradigm shift from conventional rigid machines [135]. Whereas rigid robots are limited by inflexibility, soft‐bodied systems offer innovative strategies for tasks requiring safe human‐robot interaction, smooth tactile sensing, and delicate handling of fragile objects [137]. The success of this interdisciplinary field critically depends on advanced soft materials that are not only compliant but also durable, fatigue‐resistant, and responsive, enabling the complex, recurring movements required for function [190]. Broadly, soft actuators are classified as either tethered systems, which are physically powered by external sources such as fluidic pumps or electrical cables, or untethered systems, which operate wirelessly by converting energy from environmental stimuli, such as magnetic fields, light, or temperature [191, 192, 193, 194, 195, 196, 197]. Hydrogels feature biomimetic properties like the softness and high water content of biological tissues. However, conventional homogeneous hydrogels possess fundamental trade‐offs that limit their utility in both classes. For tethered actuators, which demand mechanical robustness and high cycle life, homogeneous hydrogels are prone to stress concentration and catastrophic failure, lacking the toughness and fatigue resistance to survive repeated, high‐amplitude deformation. Conversely, for untethered robots, which require “physical intelligence” to self‐actuate, conventional hydrogels are chemically passive and lack the intrinsic stimuli‐responsive mechanisms needed to convert environmental cues into programmed motions. Phase separation provides a viable strategy to resolve these limitations. By creating heterogeneous, multiphase architectures, phase separation decouples robust mechanics from responsive function. For load‐bearing tethered components, arrested phase separation (e.g., via crystallization or salting‐out processing) can be engineered to form tough, percolating skeletons with high energy dissipation and fatigue thresholds in the kilojoule‐per‐square‐meter regime [6, 198]. Simultaneously, for untethered systems, phase separation enables the integration of intrinsically stimuli‐responsive domains, such as those exhibiting LCST or UCST behavior, to enable the material itself to encode actuation logic, modulate stiffness in situ, and convert thermal or chemical energy into complex locomotion [199, 200].

**FIGURE 17:**
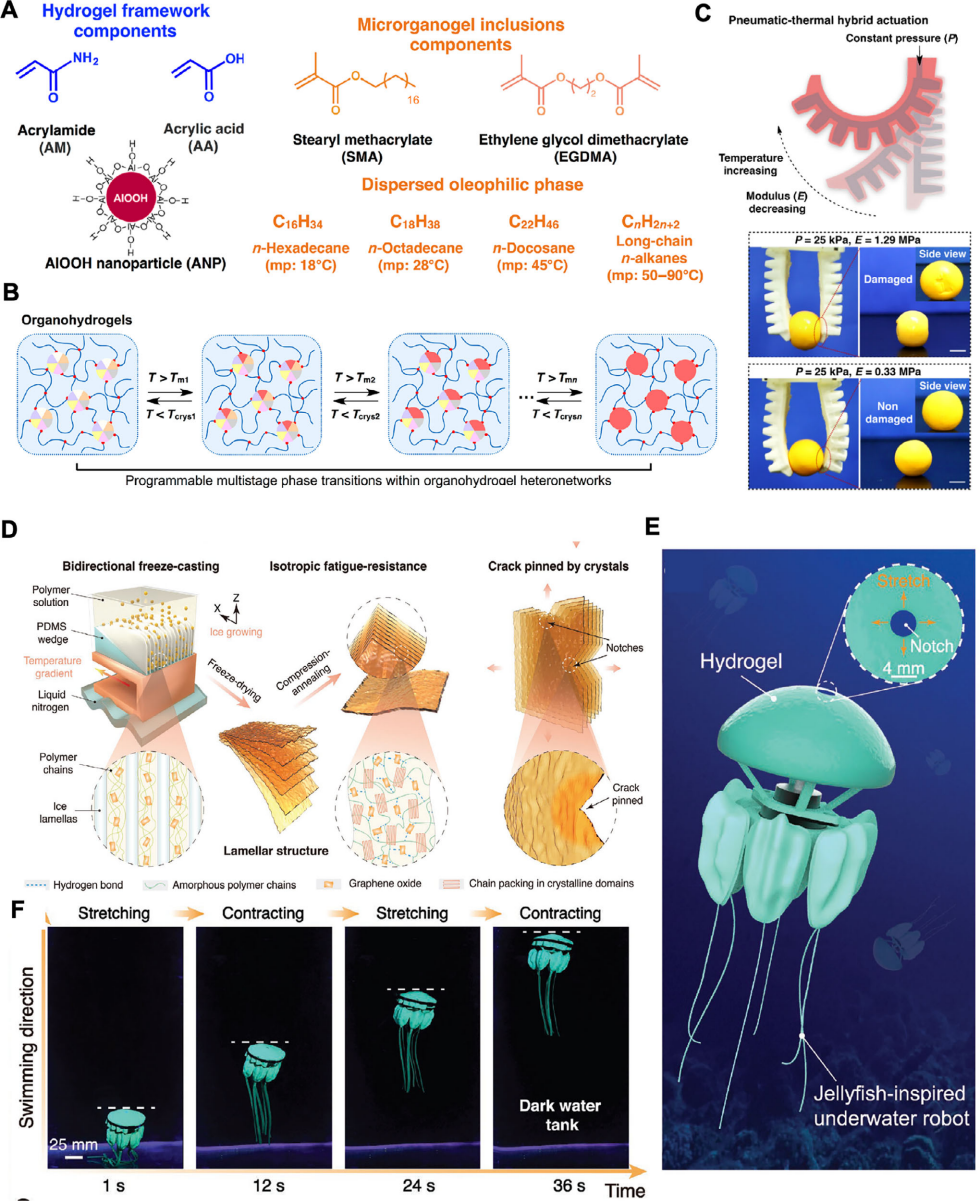
Phase‐separated hydrogel for for soft robotics and actuators. (A) The chemical components for the preparation of the multiphase organohydrogel. (B) Schematic illustration of hydrogel's programmable multiphase transition mechanism. (C) The pneumatic thermal hybrid actuation of the hydrogel‐based grid for grasping a fragile plasticine ball [164]. Reproduced with permission. Copyright 2020, American Association for the Advancement of Science. (D) Schematic illustration for fabricating PVA/GO hydrogels through the bidirectional freeze‐casting strategy. (E) Schematic of a jelly fish‐like underwater robot with PVA/GO hydrogel as the load‐bearing part. (F) The actuation process of the jellyfish‐like robot [202]. Reproduced with permission. Copyright 2022, John Wiley and Sons.

**FIGURE 18:**
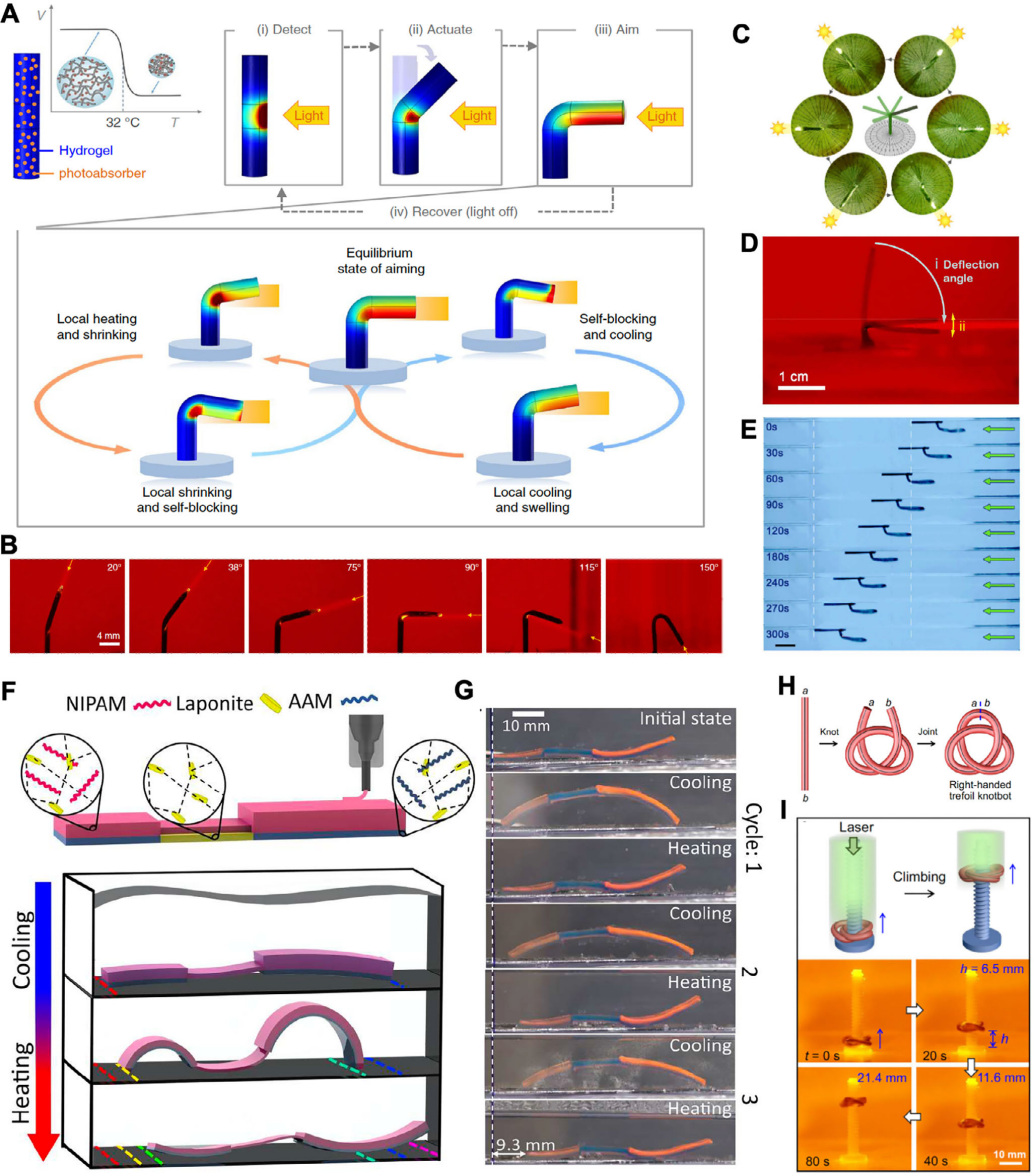
Phase‐separated hydrogel for soft robotics and actuators. (A) The design rationale of SunBOT and phototropic mechanism. (B) The submerged SunBOT autonomously aligns with laser stimuli across zenith angles ranging from 20° to 150°. (C) Top‐view snapshots capture the SunBOT continuously tracking an azimuthally revolving light source [206]. Reproduced with permission. Copyright 2019, Springer Nature. (D) Images of the OsciBots oscillation process. (E) Sequential snapshots capture the swimmer's motion under constant illumination [207]. Copyright 2019, American Association for the Advancement of Science. (F) Illustration of the fabrication process and thermal actuation of hydrogel‐based crawler. (G) Experimental snapshots capture the robot during three successive thermal cycles [197]. Reproduced with permission. Copyright 2022, American Association for the Advancement of Science. (H) Schematic of the preparation of the hydrogel knotbot. (I) A knotbot climbs along a smooth rod under constant light irradiation. Reproduced under terms of the CC‐BY license. [208] Copyright 2024, The Authors, published by Springer Nature.

A key strategy for tethered actuators is to harness temperature‐induced phase separation to achieve active, in‐situ control over mechanical properties. Zhuo et al. demonstrated this concept by fabricating complex multiphase organohydrogels [164]. This system was prepared by emulsion‐templated and in‐situ polymerization of an oil‐in‐water emulsion (Figure 17A). The aqueous phase contained hydrophilic monomers (AAM, AA), while the oil phase contained a hydrophobic monomer (stearyl methacrylate, SMA) and a modular mix of n‐alkanes (e.g., C16, C18, C28). UV gelation arrested this structure and trapped phase‐transitioning micro‐organogel inclusions within the elastic hydrogel matrix. The critical phase separation mechanism involves on‐demand modular assembly of non‐eutectic phase transition components. The different n‐alkane inclusions melted at distinct temperatures (e.g., 18°C, 35°C, 57°C), which allowed the material to exhibit stepwise or multistable mechanics (Figure 17B). This material was integrated into a pneumatic‐thermal hybrid soft gripper. At a constant pneumatic pressure, electrical heating of the gripper caused the inclusions to melt resulting in a drop of the modulus from 1.29 to 0.33 MPa (Figure 17C). This enabled adaptive grasp of a fragile plasticine ball without damage as the gripper adjusted to match the stiffness of the ball. Phase separation can also be used to fabricate tethered actuators from sustainable biopolymers. Yao and colleagues developed a hyperelastic, edible, and biodegradable soft robot from starch [201]. This work employed an antisolvent‐induced phase separation strategy. Native starch was first gelatinized in a glycerol/water (good solvent) mixture. The addition of ethanol (antisolvent) triggered phase separation and subsequent heating fixed the phase‐separated structure. The phase separation was essential for transforming brittle starch into a hyperelastic material. The antisolvent processing reconfigured the starch chains, driving the formation of dense, energy‐dissipating domains (B‐ and V‐type crystals) within a soft hydrogen‐bonded matrix. This phase separation‐induced heterogeneous structure provided the robust mechanics and high stretchability (up to 361%) required for a pneumatic actuator. A pneumatic soft gripper was successfully fabricated from the phase‐separated starch hydrogel. When pressurized, the gripper bent 55° and demonstrated gentle gripping of fragile food items like peanuts and gummies. To address the critical demand for durability in load‐bearing tethered components, a PVA/GO hydrogel with unprecedented 2D isotropic fatigue resistance was demonstrated [202]. This was achieved through bidirectional freeze‐casting (i.e., ice‐templating) to create 2D lamellar microstructures, followed by compression annealing to facilitate crystallization (Figure 18D). This phase separation‐engineered architecture yielded an extraordinary and isotropic fatigue threshold (>1500 J m^−2^). As a load‐bearing bell for a jellyfish‐inspired robot (Figure 18E), the 2D phase separation‐gel endured over 1 million actuation cycles without failure, dramatically outperforming conventional hydrogels (Figure 18F).

For untethered robots, the central challenge shifts from mechanical durability to physical intelligence – the ability of a material or system to sense, process, and respond to environmental stimuli without external wiring. Unlike tethered systems powered by pumps or wires, untethered robots must convert energy directly from a constant ambient source (e.g., heat, light, and chemicals) into sustained locomotion [135]. This requires a built‐in mechanism to spontaneously break and restore symmetry, often via a negative feedback loop [203, 204]. Phase separation provides a viable mechanism to deliver this autonomous function. Within phase‐ separated hydrogels, LCST polymers‐based networks are the most widely exploited materials for untethered actuators. PNIPAM is the canonical example, with an LCST of approximately 32°C inducing a coil‐to‐globule phase separation [139]. This polymer level transition is harnessed in PNIPAM hydrogels for macro‐actuation, where cooling keeps the network swollen, while local heating (above the LCST) triggers demixing and a rapid volume collapse to produce a contractile network and macro‐motion [205]. By incorporating photothermal absorbers (e.g., Au nanoparticles, polyaniline and reduced graphene oxide), this macro‐motion can be remotely triggered by light and actuates untethered robotic locomotion.

In a foundational demonstration, He and colleagues utilized a self‐shadowing‐enabled negative feedback loop in a PNIPAM pillar. In this case, light‐induced local heating triggers LCST phase separation and shrinkage of the illuminated side, causing the pillar to bend toward the source (Figure 18A). The bending action caused the pillar's own tip to block the light [206]. This self‐shadowing allowed the pillar to unbend and be exposed again to the light, completing the operation cycle (Figure 18B). By engineering this phase separation‐driven feedback loop, they designed a stable sunflower‐like biomimetic omnidirectional tracker (SunBots, Figure 18C) that autonomously orients toward the light source (i.e., phototropism) and an out‐of‐equilibrium OsciBots (Figure 18D). These latter systems use loosely cross‐linked gels with fast diffusion and enter perpetual oscillation under constant light irradiation. The oscillating motion was harnessed to power an all‐soft swimming robot with light‐steered phototactic capabilities (Figure 18E) [207]. Compared to locally activated actuations, achieving globally activated motions requires an asymmetric design of the actuators’ morphologies. Pantula et al. demonstrated this by 3D printing different‐sized hydrogel crawlers consisting of an active PNIPAM layer and a passive PAAM layer (Figure 18F) [197]. As the entire robot underwent heat‐cooling cycles, deswelling (heating) and swelling (cooling) occurred asynchronously between the segments. This generated an asymmetry in contact forces and broke frictional symmetry, converting a simple expansion/contraction into a unidirectional crawling motion (Figure 18G). This concept was advanced further by Zhu et al., who coupled phase separation with topology by tying a photothermal PNIPAM cylinder into a knotbot (Figure 18H) [208]. Under uniform illumination, the knot's topology creates inherent self‐shadowing at its crossings, where the illuminated top strand undergoes phase separation and rolls, which cyclically exposes the next segment to the light. This feedback cycle resulted in a continuous, self‐regulated spinning locomotion capable of rotating gears and climbing rods (Figure 18I).

#### 4.2.2 Wearable and Flexible Devices

In parallel with the implantable bioelectronics detailed in Section 4.1.2, phase‐separated hydrogels are emerging as foundational materials for a broad range of wearable and flexible systems, including on‐skin electronics, tactile interfaces for soft robotics, and smart textiles [155, 175, 209, 210]. These applications demand a unique fusion of tissue‐like compliance with high fatigue tolerance, stable conductivity, and robust interfacial adhesion in dynamic environments [211]. Phase separation provides a versatile strategy to decouple the material's mechanical response from its electronic, optical, or adhesive properties. This design freedom enables the rational engineering of materials that resolve the core trade‐offs of wearable devices [95, 212]. As a complement to the preceding discussions on in vivo devices, this section highlights how phase separation‐enabled strategies address the challenges of wearable and flexible systems with a focus on stretch‐robust conductivity and motion‐insensitive interfaces

**FIGURE 19:**
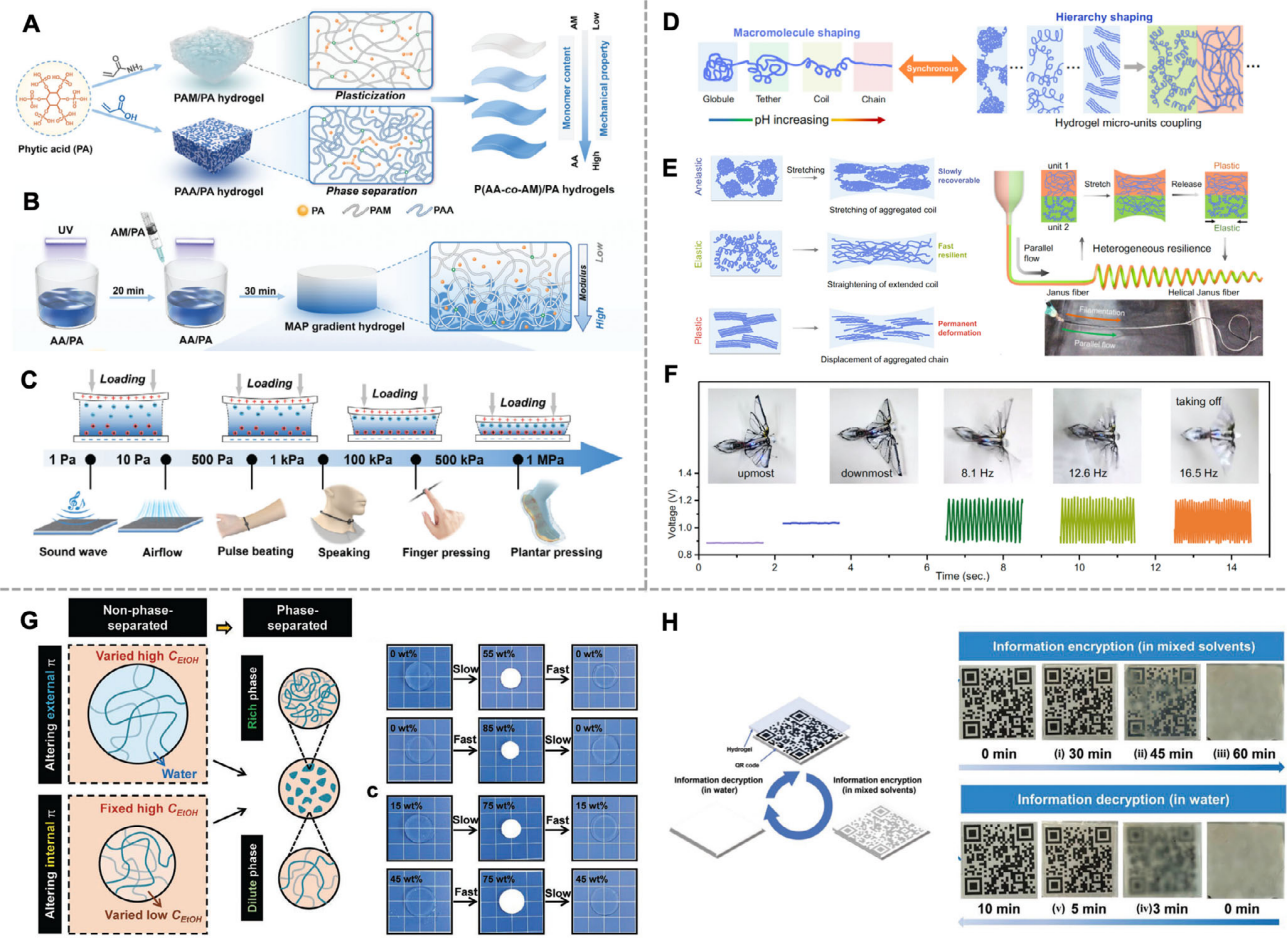
Phase‐separated hydrogel for wearable and flexible devices. (A) Illustration represents the construction of PAM/PA hydrogel and PAA/PA hydrogels and the distinct interactions of PA with different polymers. (B) Illustration showing the preparation process of gradient hydrogel via precursor solution infiltration. (C) Gradient sensing capability of MAP hydrogel for different applications [189]. Reproduced with permission. Copyright 2025, John Wiley and Sons. (D) Conformational shaping of the polyelectrolyte macromolecule based on the pH‐dependent antisolvent PS. (E) Schematic illustration of the hydrogel fiber extrusion process and the response of different macromolecule network architectures to the mechanical loading. (F) Wirelessly received sensing signals corresponding to different motion states of the robotic bird. Reproduced under terms of the CC‐BY license [213]. Copyright 2022, The Authors, published by Springer Nature. (G) Illustration represents the phase separation and transmittance changing process of PAAM in ethanol/water mixture. (H) The encryption and decryption cycle of a QR code by utilizing the phase separation behavior of PAAM hydrogel [214]. Reproduced with permission. Copyright 2022, John Wiley and Sons.

A primary challenge in e‐skin is resolving the trade‐off between sensitivity and sensing range. Phase separation provides a direct solution by enabling the creation of modulus gradients. Song et al. chemically programmed such a gradient by exploiting the distinct interaction of phytic acid (PA) with different polymers (Figure 19A) [189]. A simple precursor infiltration method was used, where a PAM/PA (polyacrylamide/phytic acid) solution was layered onto a partially polymerized PAA/PA (polyacrylic acid/phytic acid) solution. Gravity‐driven diffusion of the monomers creates a vertical concentration gradient (Figure 19B). The PA acts as a plasticizer in the PAM‐rich layer and makes it soft, while in the PAA‐rich layer, it induces microphase separation and makes it tough and reinforced. This PA‐induced separation in PAA is driven by LLPS in the precursor stage, where PA sequesters water and forces AA monomers to aggregate. This phase separation‐driven modulus gradient is the key to the sensor's performance. The soft PAM‐rich layer allows for large deformation under subtle pressures, ensuring high sensitivity, while the tough PAA‐rich layer prevents deformation saturation, enabling a wide dynamic range. The resulting pressure sensor (MAP hydrogel) achieved both high sensitivity of 9 kPa^−1^ in the low‐pressure regime and an exceptionally broad sensing range from 3.7 Pa to 1.2 MPa. This allows signal detection across multiple scales, from subtle acoustic waves and airflow to high‐magnitude plantar pressure (Figure 19C). Beyond bulk sensors, phase separation is critical for manufacturing high‐performance conductive fibers suitable for smart textiles and stretchable interconnects. Wang et al. employed a macromolecule conformational shaping process where a single‐polymer sodium polyacrylate (PANa) hydrogel fiber is spun using a pH‐dependent antisolvent phase separation method [213]. The initial pH of the dope (from 3.95 to 13.97) dictates the polymer's conformation (e.g., coiled or extended, Figure 19D). Extrusion into an antisolvent (methanol) drives phase separation and aggregation, transforming these distinct conformations into a densely entangled network. The crucial role of phase separation here is to enable extreme mechanical programming from a single material. By arresting different conformational states ranging from aggregated globules to aligned chains, the fibers can be precisely tuned to be anelastic, highly elastic, or plastic (Figure 19E). This allows for the design of sensors with tailored mechanical responses. This process yielded fibers with a modulus spanning four orders of magnitude and excellent stretchability of up to 2693%. The resulting fiber sensors can monitor large strains up to 1000% with an ultrafast response time as low as 50 ms and high durability, as demonstrated in monitoring the high‐speed wing flapping of a robotic bird (Figure 19F). To integrate high conductivity with robust mechanics, Hu et al. reported on another study utilizing continuous phase separation in a PVA/AgNW system [128]. This method combines freeze‐thawing, salting‐out, and drying‐annealing processes to induce continuous phase separation. The freeze‐thawing step creates an initial porous structure, the salting‐out processing promotes the formation of nanofibrils, and finally the drying‐annealing step drives aggregation and crystallization. This processing not only promotes the concentration and aggregation of AgNWs into interconnected, highly conductive networks but also builds a hierarchical PVA structure with high crystallinity and large crystal domains. The resulting hydrogel fibers exhibited ultrahigh conductivity (958 S cm^−1^), excellent mechanical toughness (10‐12 MJ m^−2^), and outstanding fatigue resistance, suitable for multidimensional soft electronics.

In addition to sensing applications, phase separation‐driven optical and mechanical changes can be harnessed for information encoding and device encapsulation. The reversible transparent‐to‐opaque transition of phase separation is ideal for information encryption. For example, Li et al. utilized mixed‐solvent‐induced phase separation in standard PAAm hydrogels [214]. Immersing the gel in an ethanol/water mixture triggered a reversible network collapse and turned the gel opaque (Figure 19G). The kinetics of phase separation can be regulated by controlling osmotic pressure through the tuning of the ethanol concentration, enabling time‐dependent encryption where a QR code can be programmed to be shown and erased (Figure 19H). Hou et al. achieved higher resolution using a paper‐like PVA/ poly(N‐vinylcaprolactam) (PNVCL) hydrogel [188]. In this system, chemical‐induced phase separation is locally triggered by the addition of a carboxylic‐containing solution, which forms H‐bonds with the PNVCL and makes it opaque with negligible volume change. This allows for multi‐level encryption, such as hiding permanent information written with polymer ink with temporary, self‐erasing patterns written with small‐molecule ink. Phase separation is also critical for creating robust materials for harsh environments. Jiang et al. developed a swelling‐resistant tough hydrogel via a solvent‐free bulk copolymerization of hydrophilic (AAc) and hydrophobic (LMA) monomers [215]. When this densely entangled copolymer is equilibrated in water, it undergoes strong phase separation. The resulting highly viscoelastic network, which matches the acoustic impedance of water, functions as a high‐performance underwater sound‐absorbing coating for acoustic stealth. Taken together, these strategies illustrate that by controlling phase separation, hydrogels can be precisely engineered to resolve complex trade‐offs of mechanical, electronic, and optical properties for next‐generation flexible and wearable systems.

## 5 Conclusions and Outlook

Over the past two decades, phase separation in polymer gels has emerged as a powerful and controllable design strategy for constructing soft materials with hierarchical structures and diverse functionalities. Demixing within polymer‐solvent or polymer‐polymer systems can generate regions of distinct composition, modulus, and water content. When this process is carefully tuned and arrested, the resulting cooperative domains exchange stress, energy, and mass across multiple length scales, creating a bridge between molecular design and macroscopic performance. Starting from the Flory‐Huggins and Flory‐Rehner frameworks, we highlighted how the competition between mixing enthalpy, entropy, and network elasticity determines whether a polymer solution remains homogeneous or demixes into polymer‐rich and solvent‐rich phases. By adjusting the interaction parameter (χ), cross‐link density, and polymerization kinetics, phase separation can proceed along binodal or spinodal pathways, forming either droplet‐matrix or bicontinuous morphologies. Arresting these processes through cross‐linking transforms transient instabilities into permanent architectures. For hydrogels, this strategy introduces internal length scales from nanometers to micrometers, providing structural hierarchy and multifunctionality that are unattainable in uniform networks.

The morphology emerging from phase separation governs how hydrogels resist fracture, store and release energy, and sustain repeated deformation. In particular, bicontinuous elastic‐elastic networks achieve high toughness and low hysteresis through cooperative deformation of interconnected phases, while semicrystalline and oriented architectures introduce strain‐stiffening and anisotropy reminiscent of biological tissues. Moreover, phase separation supports reversible and adaptive energy dissipation, enabling hydrogels to survive fatigue and recover their original configuration. Beyond mechanics, phase separation imparts unique responsive and multifunctional behaviors. LCST and UCST transitions exploit temperature‐dependent polymer‐solvent interactions to reversibly reorganize microdomains, producing thermally switchable stiffness, transparency, and actuation. In ionic or solvent‐driven systems, demixing creates bicontinuous channels that enhance both ion transport and water evaporation, demonstrating that ionic phase separation can directly serve electrochemical and energy‐harvesting applications. An equally important outcome is the construction of porous and perfusable architectures, which, as derived scaffolds with interconnected pores, support high cell viability, rapid migration, and lineage‐specific differentiation by coupling mechanical integrity with efficient nutrient transport. Together, these examples show that phase separation is not simply a means of reinforcing hydrogels but a unifying principle that governs their mechanical, physical, and biological functionality.

The utility of phase separation is most profoundly demonstrated in its capacity to resolve fundamental, long‐standing trade‐offs in soft materials, enabling high‐performance systems across bioadhesion, bioelectronics, tissue engineering, and soft robotics. In bioadhesion, phase separation resolves the conflict between rapid wet‐setting and long‐term durability. It enables architectures (e.g., polymer‐dense skins, dissipative skeletons) that simultaneously elevate intrinsic interfacial work and bulk energy dissipation, yielding robust seals that withstand supraphysiological pressures while mitigating off‐target adhesions. In bioelectronics, it reconciles mechanical compliance with stable charge transport. Bicontinuous morphologies decouple these properties: percolating conductive networks (e.g., PEDOT‐rich pathways) minimize bulk impedance, while stabilized, low‐swelling interfaces curb motion artifacts and signal drift, facilitating low‐threshold, chronic in vivo interfacing. In tissue engineering, it overcomes the dichotomy between mechanical integrity and functional porosity. Templating techniques like LLPS and ATPS engineer perfusable, interconnected architectures with graded mechanics. This structure is critical for coupling nutrient transport directly to cell fate decisions and promoting deep tissue integration. Finally, in soft robotics and wearable devices, it transforms passive materials into active systems. Stimuli‐responsive phase switching, triggered thermally, chemically, or by strain, provides a mechanism to dynamically program material properties, including stiffness, friction, and optical readouts, enabling robust, long‐cycle actuation and reliable on‐skin sensing.

Despite these remarkable advances, critical challenges remain before phase‐separated hydrogels can achieve the sophistication and reliability of natural systems. Most current characterization methods rely on post‐mortem imaging, which distorts hydrated morphologies and masks dynamic processes. Real‐time, high‐resolution techniques, such as operando small‐angle X‐ray scattering, cryogenic confocal microscopy, and neutron spectroscopy, are required to visualize domain evolution during deformation and under external stimuli. Moreover, most phase‐separated structures remain random. Achieving ordered, aligned, or gradient architectures through directed fields, patterned illumination, or controlled flow would enable deterministic anisotropy and reproducible multifunctionality. Finally, long‐term stability and process scalability remain major bottlenecks. Dehydration, ion leaching, and morphological coarsening degrade performance, while freeze‐casting and solvent‐exchange approaches are difficult to scale industrially. Developing continuous‐flow or additive‐manufacturing routes with real‐time phase‐separation control will be crucial for transitioning laboratory discoveries into practical materials.

Looking forward, several opportunities define the next stage of research in this field. Machine‐learning‐guided molecular design has already demonstrated its potential in identifying high‐performance underwater adhesives by predicting optimal monomer sequences and processing conditions. Expanding such data‐driven approaches to broader polymer families and linking predicted sequence descriptors to Flory‐Huggins parameters could convert empirical optimization into predictive synthesis. In parallel, additive manufacturing are emerging as a platform to pattern phase‐separated morphologies in three dimensions. Real‐time feedback between polymerization, demixing, and light or flow control may allow the spatial programming of microstructures and gradients that mimic biological tissues. Dynamic and reconfigurable phase separation is another frontier. By coupling phase boundaries to external stimuli such as light, electric fields, or magnetic gradients, researchers can achieve real‐time reconfiguration for self‐healing, adaptive robotics, or shape‐morphing systems. In the biomedical domain, biohybrid hydrogels that combine synthetic phase‐separated scaffolds with living cells offer the possibility of engineered tissues that grow, remodel, and self‐repair in situ. Likewise, porous phase‐separated architectures can provide ionic pathways for sensing, energy storage, and mass transport, integrating mechanical resilience with functional conductivity. The convergence of these directions will transform phase separation from a spontaneous process into a programmable, multifunctional design rule.

## Conflicts of Interest

M.M.S. has invested in, consults for (or is on scientific advisory boards or boards of directors) and conducts sponsored research funded by companies related to the biomaterials field; has filed patent applications related to biomaterials; and has co‐founded companies in the biomaterials field. The rest of the authors declare no conflict of interests.
